# Proton Pump Inhibitor Use and Risk of Serious Infections in Young Children

**DOI:** 10.1001/jamapediatrics.2023.2900

**Published:** 2023-08-14

**Authors:** Marion Lassalle, Mahmoud Zureik, Rosemary Dray-Spira

**Affiliations:** 1 EPI-PHARE, Epidemiology of Health Products (French National Agency for the Safety of Medicines and Health Products [ANSM], and French National Health Insurance [CNAM]), Saint-Denis, France; 2 Versailles Saint-Quentin-en-Yvelines University, Versailles, France

## Abstract

**Importance:**

Proton pump inhibitor (PPI) use may lead to infections through alteration of the microbiota or direct action on the immune system. However, only a few studies were conducted in children, with conflicting results.

**Objective:**

To assess the associations between PPI use and serious infections in children, overall and by infection site and pathogen.

**Design, Setting, and Participants:**

This nationwide cohort study was based on the Mother-Child EPI-MERES Register built from the French Health Data System (SNDS). We included all children born between January 1, 2010, and December 31, 2018, who received a treatment for gastroesophageal reflux disease or other gastric acid–related disorders, namely PPIs, histamine 2 receptor antagonists, or antacids/alginate. The index date was defined as the first date any of these medications was dispensed. Children were followed up until admission to the hospital for serious infection, loss of follow-up, death, or December 31, 2019.

**Exposure:**

PPI exposure over time.

**Main Outcomes and Measures:**

Associations between serious infections and PPI use were estimated by adjusted hazard ratios (aHRs) and 95% CIs using Cox models. PPI use was introduced as time-varying. A 30-day lag was applied to minimize reverse causality. Models were adjusted for sociodemographic data, pregnancy characteristics, child comorbidities, and health care utilization.

**Results:**

The study population comprised 1 262 424 children (median [IQR] follow-up, 3.8 [1.8-6.2] years), including 606 645 who received PPI (323 852 male [53.4%]; median [IQR] age at index date, 88 [44-282] days) and 655 779 who did not receive PPI (342 454 male [52.2%]; median [IQR] age, 82 [44-172] days). PPI exposure was associated with an increased risk of serious infections overall (aHR, 1.34; 95% CI, 1.32-1.36). Increased risks were also observed for infections in the digestive tract (aHR, 1.52; 95% CI, 1.48-1.55); ear, nose, and throat sphere (aHR, 1.47; 95% CI, 1.41-1.52); lower respiratory tract (aHR, 1.22; 95% CI, 1.19-1.25); kidneys or urinary tract (aHR, 1.20; 95% CI, 1.15-1.25); and nervous system (aHR, 1.31; 95% CI, 1.11-1.54) and for both bacterial (aHR, 1.56; 95% CI, 1.50-1.63) and viral infections (aHR, 1.30; 95% CI, 1.28-1.33).

**Conclusions and Relevance:**

In this study, PPI use was associated with increased risks of serious infections in young children. Proton pump inhibitors should not be used without a clear indication in this population.

## Key Points

**Question:**

Are proton pump inhibitors (PPIs) associated with serious infections in children?

**Findings:**

This cohort study based on data from the EPI-MERES Register in France found that PPI exposure was associated with increased risks of serious infections overall and by infection site and pathogen.

**Meaning:**

Proton pump inhibitors should not be used without a clear indication in children.

## Teaser

This cohort study examines data from French national registers for children who received treatment for gastric acid–related disorders to determine the association between use of proton pump inhibitors and serious infections in this population.

## Introduction

Proton pump inhibitors (PPIs) effectively reduce gastric acid secretion and are one of the main treatments for gastroesophageal reflux disease (GERD). In infants, GERD may be difficult to distinguish from uncomplicated gastroesophageal reflux,^1^ a physiological process involving spitting up, that affects up to 60% to 70% of infants at age 3 to 4 months and resolves spontaneously with standing and walking by 12 months.^2,3^ Uncomplicated gastroesophageal reflux does not require PPI treatment.^3^

PPI use is growing among young children in most high-income countries. In France, 6.1% of children younger than 2 years used PPIs in 2019, up from 3.6% in 2010.^4^ In the first year of life, prevalence of PPI use increased from 2.4% to 5.2% between 2005 and 2012 in New Zealand,^5^ while prescription rates were 1.9% in Sweden, 2.3% in Norway, and 4.6% in Denmark in 2020, a 3- to 5-fold increase since 2000.^6,7^

PPI use has been associated with bone fractures,^8^ acute kidney injury,^9^ allergy,^10^ asthma,^11^ and inflammatory bowel diseases^12^ in children. They are also suspected of leading to infections^13^ through pH modification or direct action on the immune system.^14^ Although young children are particularly vulnerable to infections,^15^ real-life pediatric data evaluating this risk are scarce. The aim of this nationwide cohort study was to investigate the association between PPI use and serious infections in young children.

## Methods

### Data Sources

The Mother-Child EPI-MERES Register was developed by EPI-PHARE from the French National Health Data System (SNDS) based on algorithms published in previous works.^16,17^ It includes all pregnancies managed in France since 2010. For pregnancies that resulted in delivery, the mother’s information is linked to the child data. Because of technical constraints, this cohort did not use data for same-sex twins.

The SNDS contains sociodemographic and medical information on all outpatient services reimbursed by the National Health Insurance since 2006, including prescribed drugs, health expenditures for long-term diseases, and physician visits. It also includes diagnoses related to hospital admissions and procedures performed during hospital stays. Details about the SNDS are provided in the eMethods in Supplement 1.

Under the permanent regulatory access granted to EPI-PHARE, this study did not require specific authorization from the French data protection authority (CNIL). The SNDS is a strictly anonymous database, so informed consent was not needed. This study followed the Strengthening the Reporting of Observational Studies in Epidemiology (STROBE) reporting guideline.

### Study Population and Follow-up

We included all children born between January 1, 2010, and December 31, 2018, who received their first-time treatment for GERD or other gastric acid-related disorders, namely PPIs (Anatomical Therapeutic Chemical [ATC] code A02BC), histamine 2 receptor antagonists (H2RAs; ATC code A02BA), or antacids/alginate (ATC codes A02A, A02BX13), between birth and December 31, 2019. These drugs share the same indications, so restricting the study population to children receiving these treatments at baseline would mitigate residual confounding. Only PPIs dramatically reduce gastric acid secretion^18^ and are therefore most likely to lead to infections. The index date was defined as the first date any of these medications was dispensed.

Children were excluded if they did not receive any outpatient care before the index date or if their mother did not receive any outpatient care in the year before the start of pregnancy; these criteria ensured a sufficient time window to identify comorbidities. Children were also excluded if they had a history of perinatal infection (*International Statistical Classification of Diseases and Related Health Problems, Tenth Revision *[*ICD-10*], codes P35 to P39) or other serious infection (*ICD-10* codes in eTable 1 in Supplement 1) identified during an inpatient stay before the index date.

Children were followed up until occurrence of serious infection, loss of follow-up (censored 1 year after the last date of outpatient care), death, or December 31, 2019, whichever came first.

### Exposure

The exposure of interest was PPI use over time, as measured by PPI exposure status (categorized as unexposed or exposed), history of PPI exposure (none, past, ongoing), and duration of any ongoing PPI exposure (unexposed, ≤6 months, 7-12 months, >12 months).

The duration of each PPI treatment was calculated considering that 1 tablet corresponds to 1 day of exposure. Treatment withdrawal was defined by a 90-day gap after the last day of exposure without any new PPI being dispensed. This time period was long enough to account for on-demand PPI use.^19^

We applied a 30-day lag on exposure because of the latency in the development of infection and to limit protopathic bias. In particular, protopathic bias was suspected of overestimating associations between pneumonia and PPI use.^20^

### Outcome

The outcome was the first occurrence of any serious infection requiring hospitalization (*ICD-10* codes as principal diagnosis; eTable 1 in Supplement 1). Serious infections were classified by site (digestive tract; ear, nose, and throat [ENT]; lower respiratory tract; kidneys or urinary tract; skin; musculoskeletal system; and nervous system) and by pathogen, viral or bacterial (eTable 1 in Supplement 1).

### Covariates

#### Sociodemographic and Medical Characteristics

Sociodemographic data included age, sex, and at childbirth the mother’s affiliation with complementary universal health insurance (free access to health care for people with low incomes), deprivation index, and size of the urban unit of residence.

Pregnancy and delivery characteristics were maternal age at start of pregnancy, use of assisted reproductive technology, maternity status (public, private), mode of delivery, gestational age (full-term, ≥37 weeks of pregnancy; moderate to late preterm, 32-36 weeks; very preterm, 28-31 weeks; extremely preterm, 22-27 weeks), birth weight adjusted for age and sex (*z* score^21^: severe macrosomia, >97th centile; macrosomia, >90th centile; normal weight, 10th-90th centile; small for gestational age, <10th centile; severe low weight, <3rd centile).

Maternal comorbidities, identified during pregnancy and within the 2 years before the start of pregnancy (by hospital discharge/long-term diseases diagnoses or ≥3 drugs dispensed), were diabetes (including gestational diabetes), hypertension (including preeclampsia), and obesity (eTable 2 in Supplement 1). We also identified consumption of tobacco, alcohol, or illicit substances during pregnancy.

Child comorbidities and drug exposures identified at the index date and throughout follow-up (by hospital discharge/long-term diseases diagnoses or drugs dispensed) were chronic respiratory diseases, neurological or degenerative diseases, diabetes, obesity, liver diseases, chronic kidney diseases and major congenital malformations of the urinary system, cardiovascular diseases, autoimmune diseases or other sources of potential immunosuppression (including cancer and transplantation), digestive diseases, chronic corticosteroid treatment (≥3 drugs dispensed in 6 months), and nonsteroidal anti-inflammatory drug treatment (≥1 drugs dispensed) (eTable 3 in Supplement 1).

#### Health Care Utilization and Season

For mothers, we identified exposure to preventive medications during pregnancy (ATC codes A11, A12, B03, J07). For children, within 3 months before the index date, we measured the number of outpatient visits (regardless of medical specialty), pediatric outpatient visits, drugs dispensed (except on the index date), and hospital stays. Both infections and PPI dispensings^22^ show seasonality, so we identified the season at the index date and throughout follow-up.

### Statistical Analysis

#### Main Analyses

Baseline characteristics of the children who did and did not receive PPIs during follow-up were compared using medians (IQR) and proportions and absolute standardized differences (ASD). We calculated crude incidence rates of serious infections (per 100 person-years) and their 95% CIs from a Poisson distribution. Associations between PPI use and serious infections (overall and separately by infection site and pathogen) were estimated by crude and adjusted hazard ratios (aHRs) and their 95% CIs using Cox models. The time scale was age in days. PPI use over time was introduced as time-varying, according to exposure status, history of exposure status, or duration of any ongoing exposure (eFigures 1-3 in Supplement 1). Models were adjusted for time-fixed covariates, such as sociodemographic data, pregnancy and delivery characteristics, maternal comorbidities, and health care utilization, for time-varying covariates, child comorbidities and drug exposures, and season.

In an additional analysis, past exposure was categorized according to time since PPI treatment withdrawal. In a subgroup analysis, the association between PPI exposure status over time and overall risk of serious infections was estimated separately (1) in children born very or extremely preterm or those with a comorbidity or a chronic corticosteroid treatment at baseline and (2) in those without any of these conditions.

#### Sensitivity Analyses

First, to rule out any effect of H2RAs in the association between PPI exposure over time and the risk of serious infections (although prevalence of H2RA use is extremely low in France^23^), an analysis was conducted excluding children who received H2RAs at the index date and censoring those who started H2RAs during follow-up. Second, any residual protopathic bias was assessed by (1) varying the lag time on exposure, (2) excluding children who were dispensed an antibiotic in the 3 months before the index date (assuming that recent antibiotic use could be related to a current infection), and (3) comparing the risk of serious lower respiratory tract infections in the 30 days before the index date according to the treatment initiated (PPI vs H2RA or antacid/alginate). Third, we calculated the E-values for both the association estimates and the limit of the confidence intervals closest to the null.^24,25^

#### Complementary Analyses

We measured the association between PPI exposure over time and traumatic injury (excluding fractures; *ICD-10* codes are provided in eTable 4 in Supplement 1), used as a negative control outcome.^26^ In the absence of association with such an unrelated event, a positive association between PPI use and serious infections is unlikely to reflect residual confounding. Analyses were performed using SAS version 9.4 (SAS Institute).

## Results

### Population

We identified 6 349 003 children born between January 1, 2010, and December 31, 2018, of whom 1 497 773 received a first-time PPI, H2RA, or antacid/alginate treatment before December 31, 2019. We excluded data for 38 561 children (2.6%) who did not receive outpatient care before the index date or whose mother did not receive outpatient care in the year before the start of pregnancy and 196 788 children (13.5%) with perinatal or serious infection before the index date. The study population comprised 1 262 424 children (median [IQR] follow-up, 3.8 [1.8-6.2] years), of whom 606 645 received PPIs at least once during follow-up (median [IQR] follow-up, 3.7 [1.8-6.0] years) and 655 779 did not (median [IQR] follow-up, 3.9 [1.9-6.4] years) (Figure).

**Figure.**
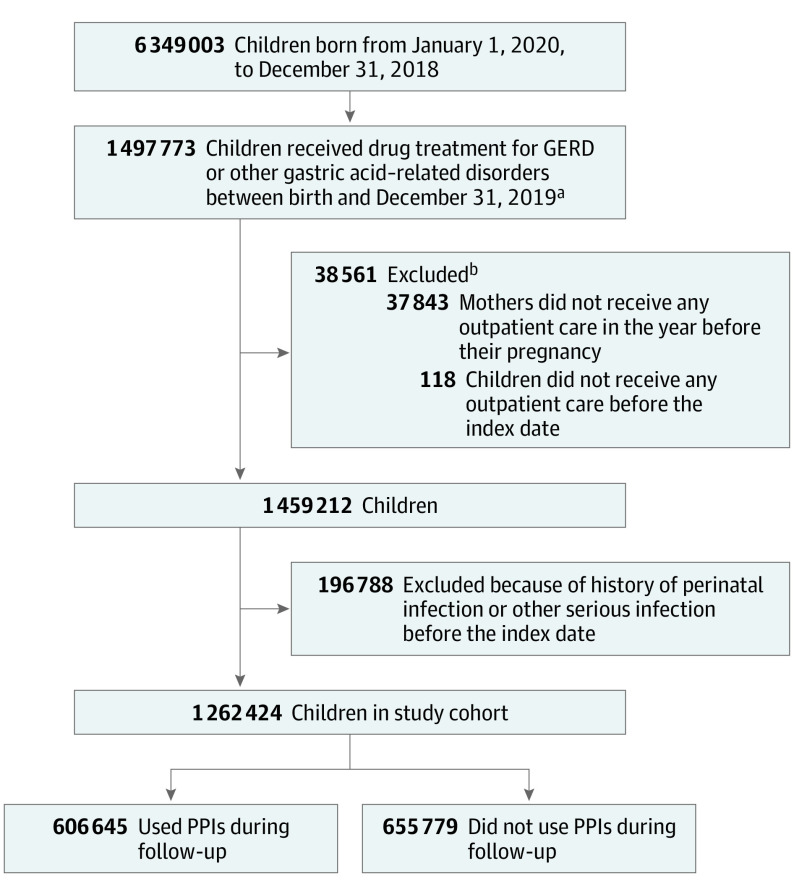
Study Flowchart GERD indicates gastroesophageal reflux disease; PPIs, proton pump inhibitors. ^a^The index date is the first date any of these drugs was dispensed. ^b^Each child may have had more than 1 exclusion criterion.

Children who did and did not receive PPIs during follow-up had a median (IQR) age of 88 (44-282) days and 82 (44-172) days at the index date, respectively (ASD, 0.22); 323 852 (53.4%) and 342 454 (52.2%) were male (ASD, 0.02), respectively. Children who received PPIs presented more frequently with comorbidities at the index date, including respiratory diseases (48 972/606 645 [8.1%] vs 23 636/655 779 [3.6%] without PPIs; ASD, 0.19), and were more likely to use corticosteroids (40 251 [6.6%] vs 19 173 [2.9%] without PPIs; ASD, 0.18) (Table 1 and eTable 5 in Supplement 1). During follow-up, children who did receive PPIs were exposed to a median (IQR) of 1 (1-1) PPI treatment episode (Table 1).

**Table 1.** Description of the Study Population

Characteristic	No. (%)		
	All (N = 1 262 424)	Received PPIs during follow-up (n = 606 645)	Did not receive PPIs during follow-up (n = 655 779)
Follow-up, median (IQR), y	3.8 (1.8-6.2)	3.7 (1.8-6.0)	3.9 (1.9-6.4)
**Sociodemographic**			
Age at index date, median (IQR), d	84 (44-210)	88 (44-282)	82 (44-172)
Sex			
Male	666 306 (52.8)	323 852 (53.4)	342 454 (52.2)
Female	596 118 (47.2)	282 793 (46.6)	313 325 (47.8)
CMUC	115 583 (9.2)	46 325 (7.6)	69 258 (10.6)
Deprivation index			
1 (Least deprived)	279 614 (22.2)	162 061 (26.7)	117 553 (17.9)
2	256 927 (20.4)	124 780 (20.6)	132 147 (20.2)
3	234 426 (18.6)	104 563 (17.2)	129 863 (19.8)
4	221 451 (17.5)	97 812 (16.1)	123 639 (18.9)
5 (Most deprived)	231 219 (18.3)	99 107 (16.3)	132 112 (20.2)
Missing	38 787 (3.1)	18 322 (3.0)	20 465 (3.1)
Urban unit			
≥200 000 Inhabitants	598 855 (47.4)	310 067 (51.1)	288 788 (44.0)
50 000-199 999 Inhabitants	153 888 (12.2)	69 943 (11.5)	83 945 (12.8)
10 000-49 999 Inhabitants	121 914 (9.7)	55 375 (9.1)	66 539 (10.2)
2000-9999 Inhabitants	140 659 (11.1)	62 930 (10.4)	77 729 (11.9)
Outside urban unit	219 151 (17.4)	96 245 (15.9)	122 906 (18.7)
Missing	27 957 (2.2)	12 085 (2.0)	15 872 (2.4)
**Pregnancy and delivery**			
Maternal age at start of pregnancy, y			
Median (IQR)	29 (26-33)	30 (26-33)	29 (26-33)
≥35	204 442 (16.2)	103 341 (17.0)	101 101 (15.4)
Assisted reproductive technology	43 923 (3.5)	23 119 (3.8)	20 804 (3.2)
Maternity status			
Public	809 006 (64.1)	382 494 (63.1)	426 512 (65.0)
Private	452 615 (35.9)	223 750 (36.9)	228 865 (34.9)
Missing	803 (0.1)	401 (0.1)	402 (0.1)
Mode of delivery			
Vaginal	997 980 (79.1)	474 852 (78.3)	523 128 (79.8)
Cesarean	263 479 (20.9)	131 324 (21.6)	132 155 (20.2)
Missing	965 (0.1)	469 (0.1)	496 (0.1)
Gestational age			
Full-term	1 175 831 (93.1)	560 990 (92.5)	614 841 (93.8)
Preterm	86 593 (6.9)	45 655 (7.5)	40 938 (6.2)
Moderate to late preterm	78 099 (6.2)	40 513 (6.7)	37 586 (5.7)
Very preterm	7181 (0.6)	4302 (0.7)	2879 (0.4)
Extremely preterm	1313 (0.1)	840 (0.1)	473 (0.1)
Birth weight			
Severe macrosomia	53 340 (4.2)	25 692 (4.2)	27 648 (4.2)
Macrosomia	79 541 (6.3)	38 025 (6.3)	41 516 (6.3)
Normal weight	941 279 (74.6)	450 899 (74.3)	490 380 (74.8)
Small weight	94 769 (7.5)	45 740 (7.5)	49 029 (7.5)
Severe low weight	51 576 (4.1)	25 823 (4.3)	25 753 (3.9)
Missing	41 919 (3.3)	20 466 (3.4)	21 453 (3.3)
**Maternal comorbidities**			
Diabetes	118 910 (9.4)	57 398 (9.5)	61 512 (9.4)
Hypertension	71 242 (5.6)	36 502 (6.0)	34 740 (5.3)
Obesity	67 802 (5.4)	30 698 (5.1)	37 104 (5.7)
Consumption of tobacco	63 999 (5.1)	27 874 (4.6)	36 125 (5.5)
Consumption of alcohol	920 (0.1)	419 (0.1)	501 (0.1)
Consumption of illicit substance	3782 (0.3)	1614 (0.3)	2168 (0.3)
**Child comorbidities and drug exposures at index date**			
Respiratory diseases	72 608 (5.8)	48 972 (8.1)	23 636 (3.6)
Neurological diseases	3870 (0.3)	2705 (0.5)	1165 (0.2)
Diabetes	133 (0.0)	80 (0.0)	53 (0.0)
Obesity	127 (0.0)	86 (0.0)	41 (0.0)
Liver diseases	477 (0.0)	345 (0.1)	132 (0.0)
Chronic kidney diseases	5452 (0.4)	2724 (0.5)	2728 (0.4)
Cardiovascular diseases	12 726 (1.0)	7678 (1.3)	5048 (0.8)
Immunosuppression	6081 (0.5)	4599 (0.8)	1482 (0.2)
Digestive diseases	63 227 (5.0)	39 497 (6.5)	23 730 (3.6)
Chronic corticosteroid treatment	59 424 (4.7)	40 251 (6.6)	19 173 (2.9)
NSAID treatment	44 313 (3.5)	28 034 (4.6)	16 279 (2.5)
Health care use in mothers			
≥3 Preventive drugs dispensed during pregnancy	769 941 (61.0)	374 016 (61.7)	395 925 (60.4)
Health care use in children, within 3 mo before index date			
≥2 Outpatient visits, all medical specialties	947 024 (75.0)	454 960 (75.0)	492 064 (75.0)
≥2 Pediatric outpatient visits	417 040 (33.0)	216 583 (35.7)	200 457 (30.6)
≥2 Drugs dispensed	740 464 (58.7)	355 287 (58.6)	385 177 (58.7)
≥1 Hospital stays	82 723 (6.6)	51 581 (8.5)	31 142 (4.8)
**PPI treatment during follow-up**			
No. of dispensings, median (IQR)		2 (1-3)	
No. of treatment episodes, median (IQR)		1 (1-1)	
Duration of treatment episodes, median (IQR), d^a^		118 (118-174)	

Abbreviations: CMUC, complementary universal health insurance; NSAID, nonsteroidal anti-inflammatory drug; PPI, proton pump inhibitor.

^a^ Including gap periods.

### Overall Risk of Serious Infections

A total of 152 055 children were newly diagnosed with a serious infection (incidence rate, 2.99 per 100 person-years; 95% CI, 2.98-3.01). Associations between serious infections and covariates are presented in eTable 6 in Supplement 1. PPI exposure over time was associated with an overall increased risk of serious infections (aHR, 1.34; 95% CI, 1.32-1.36) (Table 2).

**Table 2.** Overall Risk of Serious Infections Associated With PPI Exposure in Children

Exposure	No. of events/No. of person-years	Incidence rate (95% CI)^a^	Crude HR (95% CI)	aHR (95% CI)^b^
PPI exposure over time				
Unexposed	126 864/4 810 746	2.64 (2.62-2.65)	1 [Reference]	1 [Reference]
Exposed	25 191/271 874	9.27 (9.15-9.38)	1.42 (1.40-1.44)	1.34 (1.32-1.36)
History of PPI exposure over time				
None	82 545/2 853 971	2.89 (2.87-2.91)	1 [Reference]	1 [Reference]
Past	44 319/1 956 775	2.26 (2.24-2.29)	1.10 (1.08-1.11)	1.07 (1.06-1.09)
Ongoing	25 191/271 874	9.27 (9.15-9.38)	1.45 (1.43-1.47)	1.36 (1.34-1.38)
Duration of ongoing PPI exposure over time				
Unexposed	126 864/4 810 746	2.64 (2.62-2.65)	1 [Reference]	1 [Reference]
≤6 mo	20 718/209 875	9.87 (9.74-10.01)	1.41 (1.39-1.43)	1.34 (1.32-1.36)
7-12 mo	3491/43 000	8.12 (7.85-8.39)	1.41 (1.37-1.46)	1.33 (1.29-1.38)
>12 mo	982/18 998	5.17 (4.86-5.50)	1.65 (1.55-1.76)	1.38 (1.30-1.47)

Abbreviations: aHR, adjusted hazard ratio; PPI, proton pump inhibitor.

^a^ Per 100 person-years.

^b^ Cox models adjusted for time-fixed covariates, namely sociodemographic characteristics (age at index date, sex, complementary universal health insurance, deprivation index, size of the urban unit, calendar year at index date); pregnancy and delivery characteristics (maternal age, assisted reproductive technology, maternity status, mode of delivery, gestational age, birth weight); maternal comorbidities (diabetes, hypertension, obesity, and consumption of tobacco, alcohol, and illicit substance); health care utilization in mothers (preventive medications during pregnancy); health care utilization in children (outpatient physician visits, outpatient pediatrician visits, drugs dispensed, hospital stays); and for time-varying covariates, namely season, child comorbidities, and drug exposures (respiratory diseases, neurological disease, diabetes, obesity, liver diseases, chronic kidney diseases, cardiovascular diseases, immunosuppression, digestive diseases, chronic corticosteroid treatment, nonsteroidal anti-inflammatory drug treatment). For each categorical covariate, missing values, if any, were considered as a separate category.

Regarding history of PPI exposure, the risk of serious infections was increased among children formerly exposed to PPI (aHR, 1.07; 95% CI, 1.06-1.09), although less markedly than for ongoing exposure (Table 2).

The median (IQR) time interval between PPI withdrawal and first occurrence of a serious infection among past users was 9.7 (3.9-21.3) months. The risk of serious infections gradually decreased with increasing time elapsed since PPI treatment withdrawal (withdrawal since ≤3 months: aHR, 1.13; 95% CI, 1.10-1.16; withdrawal since >12 months: aHR, 1.03; 95% CI, 1.01-1.05) (eTable 7 in Supplement 1).

PPI exposure over time was associated with an overall increased risk of serious infections in both children born severely preterm and those with a chronic condition (aHR, 1.36; 95% CI, 1.32-1.41) and in those without any of these conditions at baseline (aHR, 1.32; 95% CI, 1.30-1.34) (eTable 8 in Supplement 1).

### Risk by Infection Site

PPI exposure over time was associated with increased risks of infections in the digestive tract (aHR, 1.52; 95% CI, 1.48-1.55), ENT sphere (aHR, 1.47; 95% CI, 1.41-1.52), lower respiratory tract (aHR, 1.22; 95% CI, 1.19-1.25), kidneys or urinary tract (aHR, 1.20; 95% CI, 1.15-1.25), musculoskeletal system (aHR, 1.17; 95% CI, 1.01-1.37), and nervous system (aHR, 1.31; 95% CI, 1.11-1.54). These risks were generally increased, although less markedly than for ongoing exposure, among children formerly exposed to PPI, with aHRs ranging from 1.01 to 1.17. The risks were globally increased regardless of duration of ongoing PPI exposure, except for musculoskeletal infections. There was no evidence of an increased risk of skin infections associated with PPI use in the main analysis (Table 3).

**Table 3.** Risk of Serious Infections Associated With PPI Exposure in Children by Site and Pathogen

Site or pathogen	No. of events/No. of person-years	Incidence rate (95% CI)^a^	Crude HR (95% CI)	aHR (95% CI)^b^
**Digestive tract**				
PPI exposure over time				
Unexposed	50 608/5 235 608	0.97 (0.96-0.98)	1 [Reference]	1 [Reference]
Exposed	9412/292 237	3.22 (3.16-3.29)	1.61 (1.57-1.65)	1.52 (1.48-1.55)
History of PPI exposure over time				
None	31 563/3 078 957	1.03 (1.01-1.04)	1 [Reference]	1 [Reference]
Past	19 045/2 156 652	0.88 (0.87-0.90)	1.10 (1.08-1.12)	1.10 (1.08-1.12)
Ongoing	9412/292 237	3.22 (3.16-3.29)	1.65 (1.61-1.69)	1.56 (1.53-1.60)
Duration of ongoing PPI exposure over time				
Unexposed	50 608/5 235 608	0.97 (0.96-0.98)	1 [Reference]	1 [Reference]
≤6 mo	7160/221 971	3.23 (3.15-3.30)	1.60 (1.56-1.64)	1.52 (1.47-1.56)
7-12 mo	1737/47 406	3.66 (3.50-3.84)	1.60 (1.53-1.68)	1.54 (1.46-1.61)
>12 mo	515/22 860	2.25 (2.07-2.46)	1.73 (1.58-1.89)	1.47 (1.35-1.61)
**ENT**				
PPI exposure over time				
Unexposed	25 052/5 375 283	0.47 (0.46-0.47)	1 [Reference]	1 [Reference]
Exposed	3700/298 771	1.24 (1.20-1.28)	1.60 (1.54-1.66)	1.47 (1.41-1.52)
History of PPI exposure over time				
None	14 835/3 149 416	0.47 (0.46-0.48)	1 [Reference]	1 [Reference]
Past	10 217/2 225 867	0.46 (0.45-0.47)	1.15 (1.12-1.18)	1.12 (1.09-1.15)
Ongoing	3700/298 771	1.24 (1.20-1.28)	1.67 (1.60-1.73)	1.53 (1.47-1.59)
Duration of ongoing PPI exposure over time				
Unexposed	25 052/5 375 283	0.47 (0.46-0.47)	1 [Reference]	1 [Reference]
≤6 mo	2657/225 391	1.18 (1.13-1.22)	1.53 (1.46-1.60)	1.44 (1.38-1.51)
7-12 mo	725/48 912	1.48 (1.38-1.59)	1.67 (1.55-1.80)	1.51 (1.40-1.63)
>12 mo	318/24 468	1.30 (1.16-1.45)	2.07 (1.85-2.31)	1.57 (1.40-1.76)
**Lower respiratory tract**				
PPI exposure over time				
Unexposed	36 607/5 260 133	0.70 (0.69-0.70)	1 [Reference]	1 [Reference]
Exposed	10 446/290 030	3.60 (3.53-3.67)	1.35 (1.32-1.39)	1.22 (1.19-1.25)
History of PPI exposure over time				
None	25 727/3 089 415	0.83 (0.82-0.84)	1 [Reference]	1 [Reference]
Past	10 880/2 170 718	0.50 (0.49-0.51)	1.12 (1.09-1.15)	1.03 (1.00-1.06)
Ongoing	10 446/290 030	3.60 (3.53-3.67)	1.38 (1.35-1.41)	1.23 (1.20-1.26)
Duration of ongoing PPI exposure over time				
Unexposed	36 607/5 260 133	0.70 (0.69-0.70)	1 [Reference]	1 [Reference]
≤6 mo	9137/220 484	4.14 (4.06-4.23)	1.34 (1.30-1.37)	1.22 (1.19-1.25)
7-12 mo	1015/46 824	2.17 (2.04-2.31)	1.32 (1.23-1.40)	1.13 (1.06-1.21)
>12 mo	294/22 723	1.29 (1.15-1.45)	2.12 (1.89-2.38)	1.47 (1.31-1.65)
**Kidneys or urinary tract**				
PPI exposure over time				
Unexposed	12 826/5 416 027	0.24 (0.23-0.24)	1 [Reference]	1 [Reference]
Exposed	2798/300 543	0.93 (0.90-0.97)	1.23 (1.18-1.29)	1.20 (1.15-1.25)
History of PPI exposure over time				
None	8831/3 167 526	0.28 (0.27-0.28)	1 [Reference]	1 [Reference]
Past	3995/2 248 502	0.18 (0.17-0.18)	1.02 (0.98-1.06)	1.01 (0.97-1.05)
Ongoing	2798/300 543	0.93 (0.90-0.97)	1.24 (1.18-1.29)	1.20 (1.15-1.26)
Duration of ongoing PPI exposure over time				
Unexposed	12 826/5 416 027	0.24 (0.23-0.24)	1 [Reference]	1 [Reference]
≤6 mo	2340/226 337	1.03 (0.99-1.08)	1.21 (1.15-1.27)	1.19 (1.13-1.25)
7-12 mo	358/49 200	0.73 (0.66-0.81)	1.24 (1.11-1.38)	1.19 (1.06-1.32)
>12 mo	100/25 006	0.40 (0.33-0.49)	1.74 (1.43-2.12)	1.44 (1.18-1.76)
**Skin**				
PPI exposure over time				
Unexposed	6127/5 469 711	0.11 (0.11-0.11)	1 [Reference]	1 [Reference]
Exposed	360/303 384	0.12 (0.11-0.13)	1.16 (1.03-1.29)	1.08 (0.97-1.21)
History of PPI exposure over time				
None	3439/3 196 852	0.11 (0.10-0.11)	1 [Reference]	1 [Reference]
Past	2688/2 272 859	0.12 (0.11-0.12)	1.09 (1.04-1.15)	1.06 (1.00-1.12)
Ongoing	360/303 384	0.12 (0.11-0.13)	1.19 (1.06-1.33)	1.11 (0.99-1.25)
Duration of ongoing PPI exposure over time				
Unexposed	6127/5 469 711	0.11 (0.11-0.11)	1 [Reference]	1 [Reference]
≤6 mo	242/227 993	0.11 (0.09-0.12)	1.09 (0.95-1.24)	1.05 (0.92-1.21)
7-12 mo	74/49 834	0.15 (0.12-0.19)	1.26 (1.00-1.59)	1.16 (0.92-1.47)
>12 mo	44/25 557	0.17 (0.13-0.23)	1.42 (1.05-1.91)	1.12 (0.83-1.51)
**Musculoskeletal system**				
PPI exposure over time				
Unexposed	2473/5 481 052	0.05 (0.04-0.05)	1 [Reference]	1 [Reference]
Exposed	203/303 579	0.07 (0.06-0.08)	1.38 (1.19-1.60)	1.17 (1.01-1.37)
History of PPI exposure over time				
None	1317/3 203 506	0.04 (0.04-0.04)	1 [Reference]	1 [Reference]
Past	1156/2 277 545	0.05 (0.05-0.05)	1.29 (1.19-1.40)	1.13 (1.04-1.23)
Ongoing	203/303 579	0.07 (0.06-0.08)	1.53 (1.31-1.78)	1.24 (1.06-1.45)
Duration of ongoing PPI exposure over time				
Unexposed	2473/5 481 052	0.05 (0.04-0.05)	1 [Reference]	1 [Reference]
≤6 mo	131/228 093	0.06 (0.05-0.07)	1.47 (1.22-1.76)	1.31 (1.09-1.57)
7-12 mo	50/49 864	0.10 (0.08-0.13)	1.27 (0.96-1.69)	1.04 (0.78-1.38)
>12 mo	22/25 623	0.09 (0.06-0.13)	1.23 (0.81-1.87)	0.91 (0.60-1.40)
**Nervous system**				
PPI exposure over time				
Unexposed	1914/5 482 847	0.03 (0.03-0.04)	1 [Reference]	1 [Reference]
Exposed	200/303 443	0.07 (0.06-0.08)	1.50 (1.27-1.76)	1.31 (1.11-1.54)
History of PPI exposure over time				
None	1030/3 204 270	0.03 (0.03-0.03)	1 [Reference]	1 [Reference]
Past	884/2 278 577	0.04 (0.04-0.04)	1.28 (1.17-1.40)	1.17 (1.06-1.28)
Ongoing	200/303 443	0.07 (0.06-0.08)	1.59 (1.35-1.88)	1.38 (1.17-1.63)
Duration of ongoing PPI exposure over time				
Unexposed	1914/5 482 847	0.03 (0.03-0.04)	1 [Reference]	1 [Reference]
≤6 mo	163/228 046	0.07 (0.06-0.08)	1.41 (1.18-1.69)	1.31 (1.09-1.57)
7-12 mo	21/49 833	0.04 (0.03-0.06)	1.62 (1.04-2.51)	1.28 (0.83-1.99)
>12 mo	16/25 564	0.06 (0.04-0.10)	2.31 (1.41-3.78)	1.35 (0.82-2.23)
**Bacterial pathogen**				
PPI exposure over time				
Unexposed	24 715/5 386 573	0.46 (0.45-0.46)	1 [Reference]	1 [Reference]
Exposed	3177/299 527	1.06 (1.02-1.10)	1.78 (1.71-1.85)	1.56 (1.50-1.63)
History of PPI exposure over time				
None	13 963/3 156 779	0.44 (0.44-0.45)	1 [Reference]	1 [Reference]
Past	10 752/2 229 794	0.48 (0.47-0.49)	1.18 (1.15-1.21)	1.13 (1.10-1.16)
Ongoing	3177/299 527	1.06 (1.02-1.10)	1.88 (1.80-1.96)	1.64 (1.57-1.71)
Duration of ongoing PPI exposure over time				
Unexposed	24 715/5 386 573	0.46 (0.45-0.46)	1 [Reference]	1 [Reference]
≤6 mo	2148/226 012	0.95 (0.91-0.99)	1.67 (1.60-1.76)	1.53 (1.45-1.60)
7-12 mo	702/49 091	1.43 (1.33-1.54)	1.88 (1.74-2.03)	1.63 (1.51-1.77)
>12 mo	327/24 425	1.34 (1.20-1.49)	2.29 (2.05-2.55)	1.63 (1.46-1.82)
**Viral pathogen**				
PPI exposure over time				
Unexposed	58 833/5 141 632	1.14 (1.14-1.15)	1 [Reference]	1 [Reference]
Exposed	14 598/285 310	5.12 (5.03-5.20)	1.38 (1.36-1.41)	1.30 (1.28-1.33)
History of PPI exposure over time				
None	40 432/3 025 875	1.34 (1.32-1.35)	1 [Reference]	1 [Reference]
Past	18 401/2 115 757	0.87 (0.86-0.88)	1.07 (1.05-1.09)	1.04 (1.02-1.06)
Ongoing	14 598/285 310	5.12 (5.03-5.20)	1.40 (1.37-1.43)	1.32 (1.29-1.34)
Duration of ongoing PPI exposure over time				
Unexposed	58 833/5 141 632	1.14 (1.14-1.15)	1 [Reference]	1 [Reference]
≤6 mo	12 425/217 685	5.71 (5.61-5.81)	1.38 (1.35-1.41)	1.31 (1.28-1.34)
7-12 mo	1743/45 728	3.81 (3.64-3.99)	1.36 (1.29-1.43)	1.27 (1.21-1.33)
>12 mo	430/21 897	1.96 (1.79-2.16)	1.59 (1.45-1.75)	1.28 (1.17-1.41)

Abbreviations: aHR, adjusted hazard ratio; ENT, ear, nose, and throat; PPI, proton pump inhibitor.

^a^ Per 100 person-years.

^b^ Cox models adjusted for time-fixed covariates, namely sociodemographic characteristics (age at index date, sex, complementary universal health insurance, deprivation index, size of the urban unit, calendar year at index date); pregnancy and delivery characteristics (maternal age, assisted reproductive technology, maternity status, mode of delivery, gestational age, birth weight); maternal comorbidities (diabetes, hypertension, obesity, and consumption of tobacco, alcohol, and illicit substance); health care utilization in mothers (preventive medications during pregnancy); health care utilization in children (outpatient physician visits, outpatient pediatrician visits, drugs dispensed, hospital stays); and for time-varying covariates, namely season, child comorbidities, and drug exposures (respiratory diseases, neurological disease, diabetes, obesity, liver diseases, chronic kidney diseases, cardiovascular diseases, immunosuppression, digestive diseases, chronic corticosteroid treatment, nonsteroidal anti-inflammatory drug treatment). For each categorical covariate, missing values, if any, were considered as a separate category.

### Risk by Pathogen

PPI exposure over time was associated with increased risks of bacterial (aHR, 1.56; 95% CI, 1.50-1.63) and viral infections (aHR, 1.30; 95% CI, 1.28-1.33). The risks were increased, although less markedly than for ongoing exposure, among children formerly exposed to PPI. The risks were also increased regardless of duration of ongoing PPI exposure (Table 3).

### Sensitivity and Complementary Analyses

After exclusion of children who received H2RAs at the index date, the overall risk of serious infections associated with PPI exposure over time remained unchanged (aHR, 1.34; 95% CI, 1.31-1.36) (eTable 9 in Supplement 1).

Higher risks of serious infections associated with PPI exposure over time were observed with 0-day or 7-day lags than with 30-day or 60-day lags, except for lower respiratory tract infections, for which the risks were almost unchanged, regardless of lag. Associations persisted when using a 60-day lag, except for musculoskeletal infections (eTable 10 in Supplement 1). Exclusion of children who were dispensed an antibiotic in the 3 months before the index date did not substantially change the results (eTable 10 in Supplement 1). We did not observe an increased risk of preexisting lower respiratory tract infections associated with PPI initiation at the index date (aHR, 0.91; 95% CI, 0.87-0.94) (eTable 11 in Supplement 1).

The E-value for the association between PPI exposure over time and the overall risk of serious infections was 2.01 for the central value of the estimate and 1.97 for the lower bound of the confidence interval. The lowest E-values were found for musculoskeletal infections (eTable 12 in Supplement 1).

We did not find an association between PPI use and traumatic injury (Table 4).

**Table 4.** Risk of Traumatic Injuries (Excluding Fractures) Associated With PPI Exposure in Children

Exposure	No. of events/No. of person-years	Incidence rate (95% CI)^a^	Crude HR (95% CI)	aHR (95% CI)^b^
PPI exposure over time				
Unexposed	14 255/5 429 807	0.26 (0.26-0.27)	1 [Reference]	1 [Reference]
Exposed	1106/301 856	0.37 (0.35-0.39)	0.96 (0.90-1.02)	0.96 (0.90-1.02)
History of PPI exposure over time				
None	8927/3 173 180	0.28 (0.28-0.29)	1 [Reference]	1 [Reference]
Past	5328/2 256 627	0.24 (0.23-0.24)	0.93 (0.90-0.97)	0.94 (0.91-0.98)
Ongoing	1106/301 856	0.37 (0.35-0.39)	0.94 (0.88-1.01)	0.94 (0.88-1.00)
Duration of ongoing PPI exposure over time				
Unexposed	14 255/5 429 807	0.26 (0.26-0.27)	1 [Reference]	1 [Reference]
≤6 mo	788/226 907	0.35 (0.32-0.37)	0.93 (0.86-1.00)	0.93 (0.86-1.01)
7-12 mo	225/49 616	0.45 (0.40-0.52)	1.01 (0.88-1.16)	1.01 (0.88-1.16)
>12 mo	93/25 334	0.37 (0.30-0.45)	1.13 (0.92-1.38)	1.01 (0.83-1.25)

Abbreviations: aHR, adjusted hazard ratio; ASD, absolute standardized differences; NSAID, nonsteroidal anti-inflammatory drug; PPI, proton pump inhibitor.

^a^ Per 100 person-years.

^b^ Cox models adjusted for time-fixed covariates, namely sociodemographic characteristics (age at index date, sex, complementary universal health insurance, deprivation index, size of the urban unit, calendar year at index date); pregnancy and delivery characteristics (maternal age, assisted reproductive technology, maternity status, mode of delivery, gestational age, birth weight); maternal comorbidities (diabetes, hypertension, obesity, and consumption of tobacco, alcohol, and illicit substance); health care utilization in mothers (preventive medications during pregnancy); health care utilization in children (outpatient physician visits, outpatient pediatrician visits, drugs dispensed, hospital stays); and for time-varying covariates, namely season, child comorbidities, and drug exposures (respiratory diseases, neurological disease, diabetes, obesity, liver diseases, chronic kidney diseases, cardiovascular diseases, immunosuppression, digestive diseases, chronic corticosteroid treatment, nonsteroidal anti-inflammatory drug treatment). For each categorical covariate, missing values, if any, were considered as a separate category.

## Discussion

To our knowledge, this is the first study investigating the risk of serious infections associated with PPI use in young children for various sites and pathogens. Our results showed increased risks of serious infections overall but also risk of infections in the digestive tract, ENT sphere, lower respiratory tract, kidneys or urinary tract, and nervous system infections and both bacterial and viral infections.

Gastric acidity constitutes a fundamental host defense by killing various ingested pathogens. By increasing gastric pH, PPIs alter the gastric microbiota, thereby promoting enteric infections.^14,27^ The composition of the microbiota undergoes major changes during infancy, especially in preterm infants.^28^ Therefore, PPI exposure during this period could have a notable impact. PPI use may lead to respiratory infections via microaspiration of gastric fluid enriched in bacteria^29^ or via the gut-lung axis.^30^ PPIs may also directly affect multiple functions of the immune system. Specifically, they could interfere with various neutrophil functions.^14^

In children, 2 meta-analyses based on the results of up to 6 studies found an increased risk of *Clostridioides difficile* infection associated with PPI use.^31,32^ Three pediatric studies examined the risk of respiratory tract infections in children, with conflicting results^20,33,34^: PPI use was associated with a doubled risk in 1 cohort study,^33^ whereas the results of 2 studies did not support an association.^20,34^

One study showed a 2-fold increased risk of central nervous system infections associated with PPI in adults.^35^ Two studies have investigated the risk of urinary tract or skin infections, also in adults: one found no association for these sites^36^ while the other showed increased risks in PPI users, which was attributed to residual confounding.^37^ The risk of musculoskeletal infections has not been studied to our knowledge.

Our findings suggest that PPI exposure increases the risk of serious infections in both children with and without a history of severe prematurity or chronic illness. This raises particular concerns for children with chronic conditions such as neurological impairment, who are especially likely to require long-term maintenance treatment with PPIs.^38^ Moreover, the increased risk of serious infections, although gradually decreasing after PPI discontinuation, persisted during several months among past PPI users. These findings are consistent with those of a previous study.^33^ However, additional research is needed to better identify high-risk populations and further investigate the effect of dose, duration, and persistence of risk after treatment withdrawal.

### Strengths and Limitations

This study has several strengths. First, the SNDS contains comprehensive data on the entire French population, which ensures adequate power and prevents selection bias. Second, the SNDS is a potent tool for identifying serious infections, with positive predictive values of 97% overall and 98% regarding the infection site.^39^ Nevertheless, to our knowledge, no study to date has evaluated the performance of these data in accurately identifying pathogens. Third, models were adjusted for health care utilization to minimize surveillance bias.^40^ Fourth, several sensitivity analyses were performed, suggesting that protopathic bias is unlikely to fully explain the associations, even for lower respiratory tract infections.

This study also has limitations. First, the SNDS does not provide information on indication for treatment. Therefore, we could not distinguish children experiencing GERD from those inappropriately treated for uncomplicated gastroesophageal reflux. PPIs are often prescribed off-label for this indication.^2^ However, their efficacy on crying and irritability, vomiting and regurgitation, or even signs and symptoms of GERD (feeding refusal, chronic cough, or arching) is not demonstrated.^1,41^ Second, information was not available on breastfeeding or social interactions. Nevertheless, analyses were adjusted for other notable risk factors for infections, such as maternal comorbidities, prematurity, low birth weight, or cesarean delivery,^42^ which is a novelty compared with previous studies conducted in young children.^20,31,32,33,34^ In addition, we showed that residual confounding tended to be minimized, through calculation of E-values and use of a negative control outcome. Third, PPI use during hospital stays or over-the-counter use is not identifiable in the SNDS but is limited. In 2015, 92% of all PPI boxes sold in France were delivered to outpatients, of which 97% were obtained with a prescription^43^; moreover, pediatric formulations are available by prescription only.

## Conclusions

In this study, increased risk of serious infections was associated with PPI use in young children, overall and for various sites and pathogens. In this population, PPIs should not be used without a clear indication.

## Notes

Supplement 1. **eMethods.** Presentation of the SNDS databases
eReferences
**eFigure 1.** PPI use over time defined as time-varying according to exposure status: examples **eFigure 2.** PPI use over time defined as time-varying according to history of exposure: examples **eFigure 3.** PPI use over time defined as time-varying according to duration of any ongoing exposure: examples **eTable 1.** List of ICD-10 codes used to identify serious infections **eTable 2.** List of codes used to identify maternal comorbidities **eTable 3.** List of codes used to identify child comorbidities and drug exposures **eTable 4.** List of ICD-10 codes used to identify traumatic injuries, excluding fractures **eTable 5.** Description of the study population at baseline **eTable 6.** Overall risk of serious infections associated with covariates **eTable 7.** Overall risk of serious infections associated with history of PPI exposure over time in children, distinguishing past PPI users according to time since PPI treatment withdrawal **eTable 8.** Overall risk of serious infections associated with proton pump inhibitor exposure over time in children, separately in children born very/extremely preterm or with a chronic comorbiditya, and in those without any of these conditions at baseline **eTable 9.** Overall risk of serious infections associated with PPI exposure over time in children, excluding H2RA users at baseline **eTable 10.** Risk of serious infections associated with proton pump inhibitor exposure over time in children, overall, by site and pathogen – Assessment of protopathic bias **eTable 11.** Risk of serious lower respiratory tract infections in the 30 days before index date according to the treatment initiated at index date (PPI vs. H2RA or antacid/alginate) **eTable 12.** E-values calculated for the association between PPI exposure over time and serious infections in children, overall, and by site and pathogen

Supplement 2. Data sharing statement
