# Supplementary material for: Proton Pump Inhibitor Use and Risk of Serious Infections in Young Children
Source: JAMA Pediatr. 2023 Aug 14;177(10):1028–38. doi: 10.1001/jamapediatrics.2023.2900 (PMC10425862; doi:10.1001/jamapediatrics.2023.2900)
Supplement: Supplement 1. — eMethods. Presentation of the SNDS databases eReferences eFigure 1. PPI use over time defined as time-varying according to exposure status: examples eFigure 2. PPI use over time defined as time-varying according to history of exposure: examples eFigure 3. PPI use over time defined as time-varying according to duration of any ongoing exposure: examples eTable 1. List of ICD-10 codes used to identify serious infections eTable 2. List of codes used to identify maternal comorbidities eTable 3. List of codes used to identify child comorbidities and drug exposures eTable 4. List of ICD-10 codes used to identify traumatic injuries, excluding fractures eTable 5. Description of the study population at baseline eTable 6. Overall risk of serious infections associated with covariates eTable 7. Overall risk of serious infections associated with history of PPI exposure over time in children, distinguishing past PPI users according to time since PPI treatment withdrawal eTable 8. Overall risk of serious infections associated with proton pump inhibitor exposure over time in children, separately in children born very/extremely preterm or with a chronic comorbiditya, and in those without any of these conditions at baseline eTable 9. Overall risk of serious infections associated with PPI exposure over time in children, excluding H2RA users at baseline eTable 10. Risk of serious infections associated with proton pump inhibitor exposure over time in children, overall, by site and pathogen – Assessment of protopathic bias eTable 11. Risk of serious lower respiratory tract infections in the 30 days before index date according to the treatment initiated at index date (PPI vs. H2RA or antacid/alginate) eTable 12. E-values calculated for the association between PPI exposure over time and serious infections in children, overall, and by site and pathogen [file jamapediatr-e232900-s001.pdf]

Supplemental Online Content

Lassalle M, Zureik M, Dray-Spira R. Proton pump inhibitor use and risk of serious infections in young children. *JAMA Pediatr*. Published online August 14, 2023. doi:10.1001/jamapediatrics.2023.2900

eMethods. Presentation of the SNDS databases

eReferences

eFigure 1. PPI use over time defined as time-varying according to exposure status: examples

eFigure 2. PPI use over time defined as time-varying according to history of exposure: examples

eFigure 3. PPI use over time defined as time-varying according to duration of any ongoing exposure: examples

eTable 1. List of ICD-10 codes used to identify serious infections

eTable 2. List of codes used to identify maternal comorbidities

eTable 3. List of codes used to identify child comorbidities and drug exposures

eTable 4. List of ICD-10 codes used to identify traumatic injuries, excluding fractures

eTable 5. Description of the study population at baseline

eTable 6. Overall risk of serious infections associated with covariates

eTable 7. Overall risk of serious infections associated with history of PPI exposure over time in children, distinguishing past PPI users according to time since PPI treatment withdrawal

eTable 8. Overall risk of serious infections associated with proton pump inhibitor exposure over time in children, separately in children born very/extremely preterm or with a chronic comorbidity[a], and in those without any of these conditions at baseline

eTable 9. Overall risk of serious infections associated with PPI exposure over time in children, excluding H2RA users at baseline

eTable 10. Risk of serious infections associated with proton pump inhibitor exposure over time in children, overall, by site and pathogen – Assessment of protopathic bias

eTable 11. Risk of serious lower respiratory tract infections in the 30 days before index date according to the treatment initiated at index date (PPI vs. H2RA or antacid/alginate)

eTable 12. E-values calculated for the association between PPI exposure over time and serious infections in children, overall, and by site and pathogen

This supplemental material has been provided by the authors to give readers additional information about their work.

© 2023 Lassalle M et al. *JAMA Pediatrics.*

eMethods. Presentation of the SNDS databases

The French National Health Data System (Système National des Données de Santé, SNDS) records detailed information on individual health insurance claims for inpatient and outpatient care, from both the SNIIRAM (Système National d'Information Inter-Régime de l'Assurance Maladie, National Health Insurance claims information system) and the PMSI (Programme de Médicalisation des Systèmes d'Information, national hospital discharge database).

The database contains data on all outpatient services reimbursed by the National Health Insurance, including drugs (coded according to the Anatomical Therapeutic Chemical Classification System [ATC][1]), physician visits, and laboratory tests, but does not provide information on medical indications, which can be deduced from other data. Patients with costly chronic diseases (LTD: long-term diseases), such as cancer, are 100% reimbursed for their health expenditure, and the diagnosis is recorded (coded according to the International Classification of Diseases, Tenth Revision [ICD-10][2]).

The database also contains the procedures performed during all hospital stays, and the principal (DP), related (DR), and associated (DA) diagnoses (coded according to ICD-10). The DP corresponds to the diseases justifying admission to hospital. In order to be recorded as a hospitalization diagnosis in the database (DP, DR, or DA), the diagnosis therefore had to have an impact on medical care. Procedures are coded according to the French medical classification of clinical procedures (CCAM, Classification commune des actes médicaux[3]). Drugs used during hospital stays are not available, except for certain expensive drugs (such as some types of cancer chemotherapy). Data available for hospital stays are admission date, discharge date, and procedure date.

About 99% of the population living in France (67 million people) are covered by one of the various national health insurance scheme. Each national health insurance scheme reimburses almost all medical care at the same rate for both public and private care. Persons with limited income or LTD are 100% reimbursed.

SNDS data are commonly used for non-interventional studies in various conditions. More than 400 published studies have used these databases[4]. These comprehensive national databases are powerful tools for assessing the prevalence of rare events. As the primary purpose of these databases is financial, any false statements constitute serious frauds, liable to legal proceedings, which also tends to ensure the accuracy of the data recorded. Furthermore, quality controls and audits are performed before transmission to National Health Insurance (CNAM, Caisse Nationale de l'Assurance Maladie), mainly for processing of aberrant or missing data.

eReferences

1. WHO Collaborating Centre for Drug Statistics Methodology. WHOCC - ATC/DDD Index. Accessed March 12, 2021. https://www.whocc.no/atc_ddd_index/

2. World Health Organization. *The ICD-10 Classification of Mental and Behavioural Disorders: Clinical Descriptions and Diagnostic Guidelines*. World Health Organization; 1992.

3. Ministère de la Santé, de la Jeunesse et des Sports. Classification commune des actes médicaux. Bull Off. 2007;(2007/3 bis). Accessed March 12, 2021. https://solidarites-sante.gouv.fr/IMG/pdf/bo0703.pdf

4. Tuppin P, Rudant J, Constantinou P, et al. Value of a national administrative database to guide public decisions: From the système national d'information interrégimes de l'Assurance Maladie (SNIIRAM) to the système national des données de santé (SNDS) in France. *Rev Epidemiol Sante Publique*. 2017;65 Suppl 4:S149-S167. doi:10.1016/j.respe.2017.05.004

© 2023 Lassalle M et al. *JAMA Pediatrics.*

eFigures

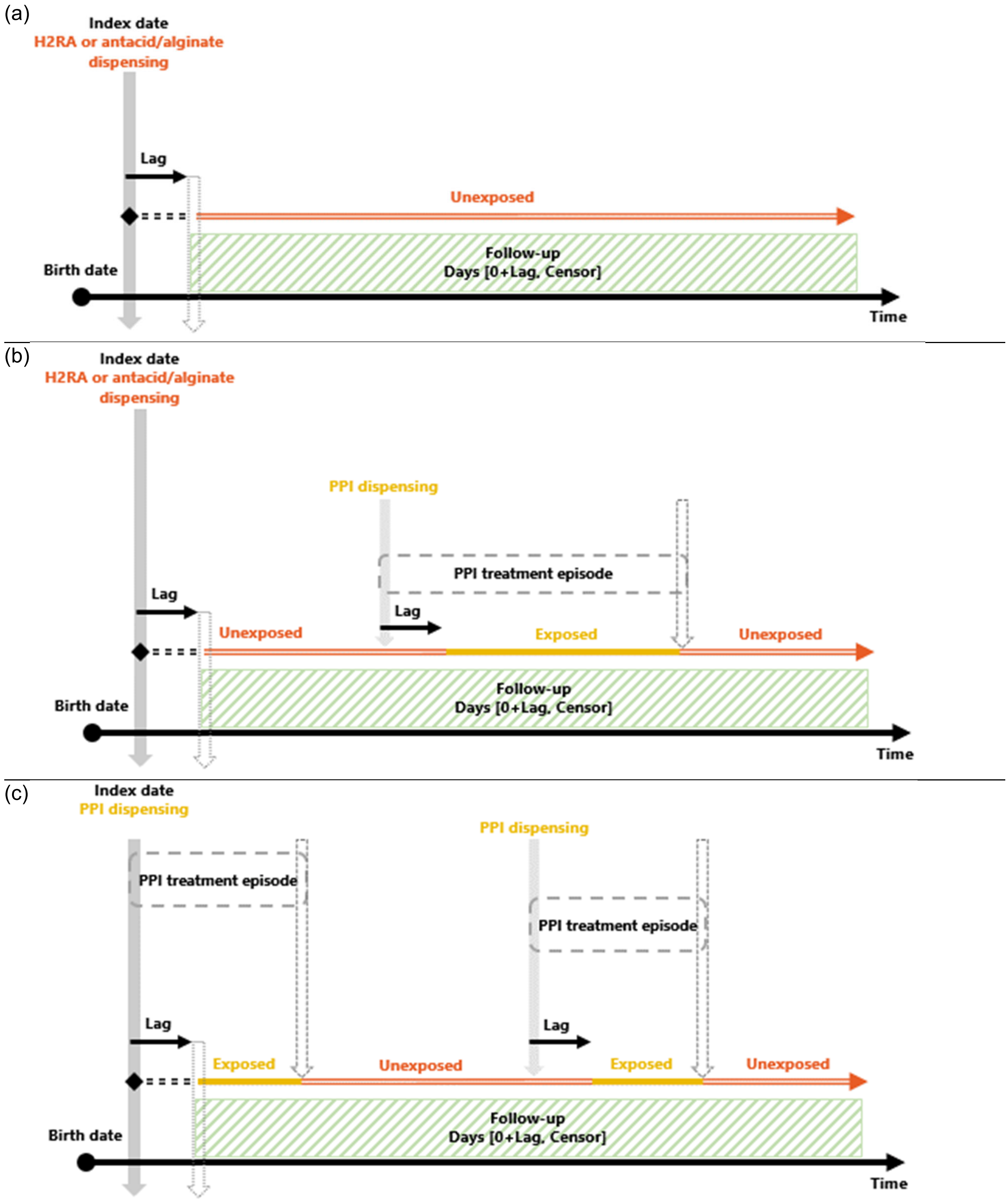

eFigure 1. PPI use over time defined as time-varying according to exposure status – Examples

© 2023 Lassalle M et al. *JAMA Pediatrics.*

eFigure 1

Index date: first dispensing of a drug treatment for reflux or other gastric acid-related disorders, namely proton pump inhibitor (PPI), histamine-2-receptor antagonist ([H2RA], or antacid/alginate.

PPI treatment episode: one or several PPI dispensings, with (at most) 90-day gaps between two consecutive dispensings.

Follow-up: children were followed from the index date (day 0)+lag until occurrence of serious infection, loss to follow-up (censored 1 year after the last identified date of outpatient care), death, or December 31, 2019, whichever came first.

(a) In this example, the child was dispensed an H2RA or antacid/alginate at index date, and did not receive any PPI dispensing during follow-up. Thus, sThus, she/heexclusively contributed as unexposed person-time.

(b) In this example, the child was dispensed an H2RA or antacid/alginate at index date, then dispensed PPI in a single PPI treatment episode during follow-up. Thus, she/he contributed successively as: unexposed person-time (from the start of the follow-up to the start of PPI treatment episode+lag), exposed person-time (from the start of PPI treatment episode+lag to the end of PPI treatment episode), then unexposed person-time (from the end of PPI treatment episode to the end of follow-up).

(c) In this example, the child was dispensed a PPI at index date. In total, during follow-up, she/he received two PPI treatment episodes. Thus, she/he contributed successively as exposed person-time, unexposed person-time, exposed person-time, then unexposed person-time.

© 2023 Lassalle M et al. *JAMA Pediatrics.*

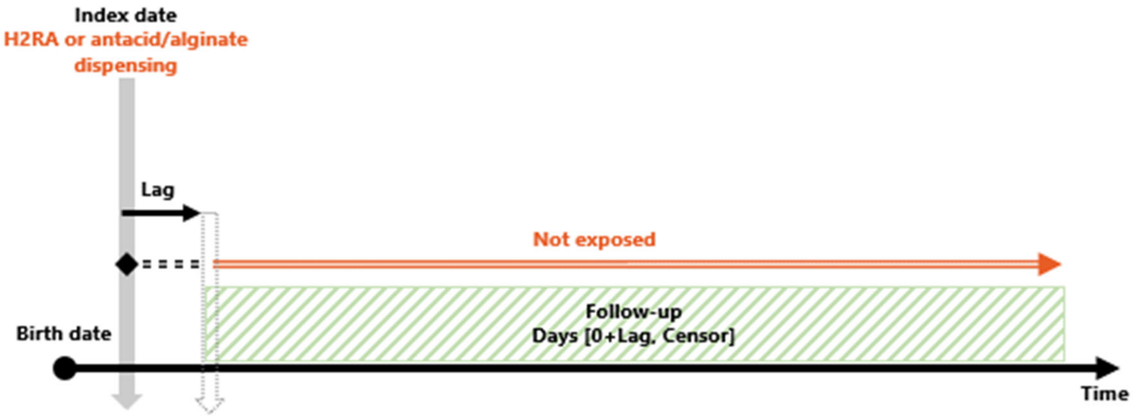

(b)

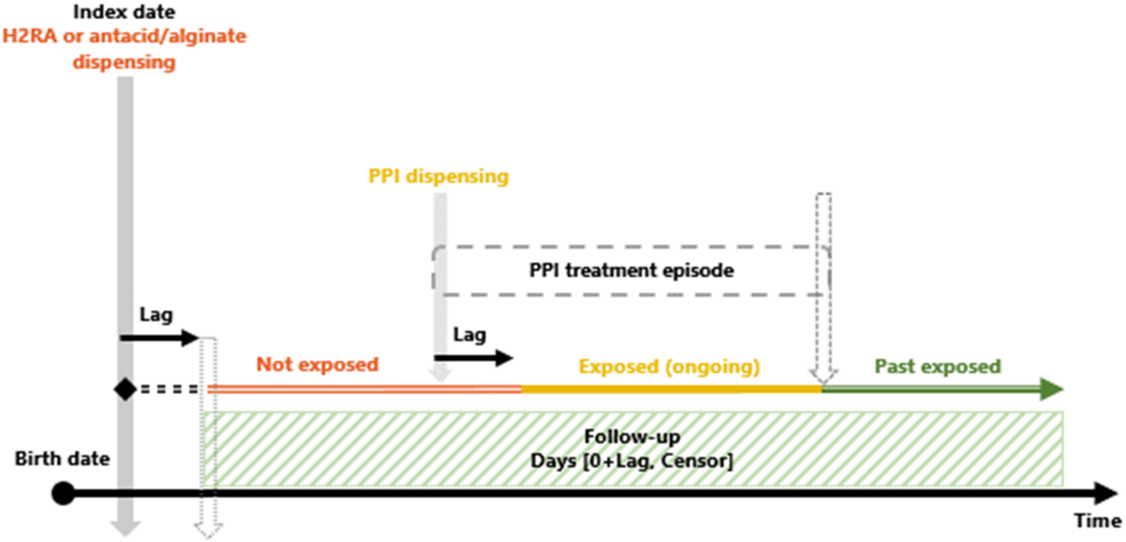

(c)

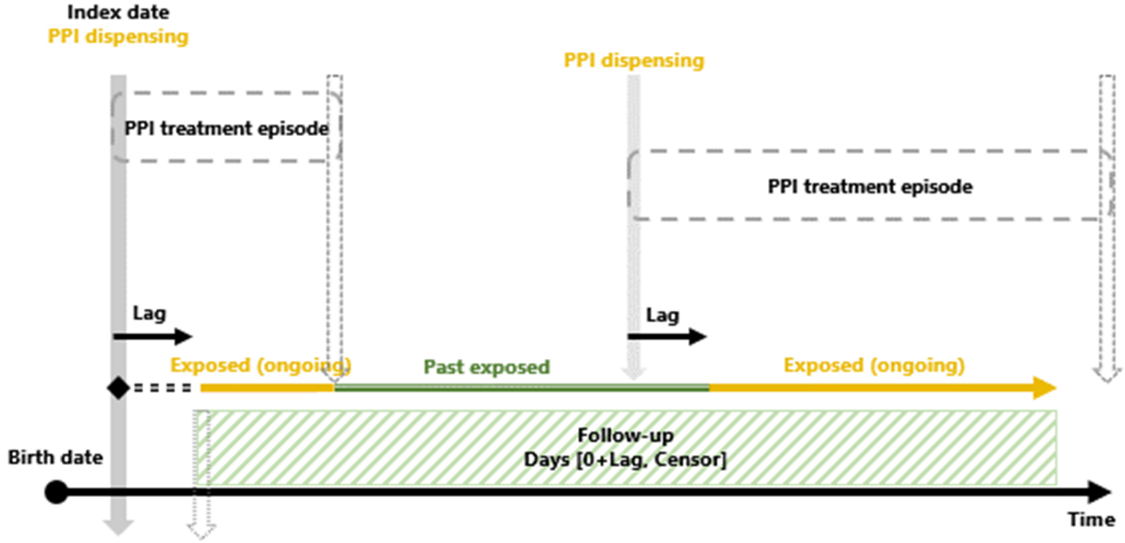

eFigure 2. PPI use over time defined as time-varying according to history of exposure – Examples

© 2023 Lassalle M et al. *JAMA Pediatrics.*

eFigure 2

Index date: first dispensing of a drug treatment for reflux or other gastric acid-related disorders, namely proton pump inhibitor (PPI), histamine-2-receptor antagonist ([H2RA], or antacid/alginate.

PPI treatment episode: one or several PPI dispensings, with (at most) 90-day gaps between two consecutive dispensings.

Follow-up: Children were followed from the index date (day 0)+lag until occurrence of serious infection, loss to follow-up (censored 1 year after the last identified date of outpatient care), death, or December 31, 2019, whichever came first.

(a) In this example, the child was dispensed an H2RA or antacid/alginate at index date, and did not receive any PPI dispensing during follow-up. Thus, she/he exclusively contributed as not exposed person-time.

(b) In this example, the child was dispensed an H2RA or antacid/alginate at index date, then dispensed PPI in a single PPI treatment episode during follow-up. Thus, she/he contributed successively as: not exposed person-time (from the start of the follow-up to the start of PPI treatment episode+lag), ongoing exposed person-time (from the start of PPI treatment episode+lag to the end of PPI treatment episode), then past exposed person-time (from the end of PPI treatment episode to the end of follow-up).

(c) In this example, the child was dispensed a PPI at index date. In total, during follow-up, he/she received two PPI treatment episodes. Thus, she/he contributed successively as ongoing exposed person-time, past exposed person-time, then ongoing exposed person-time.

© 2023 Lassalle M et al. *JAMA Pediatrics.*

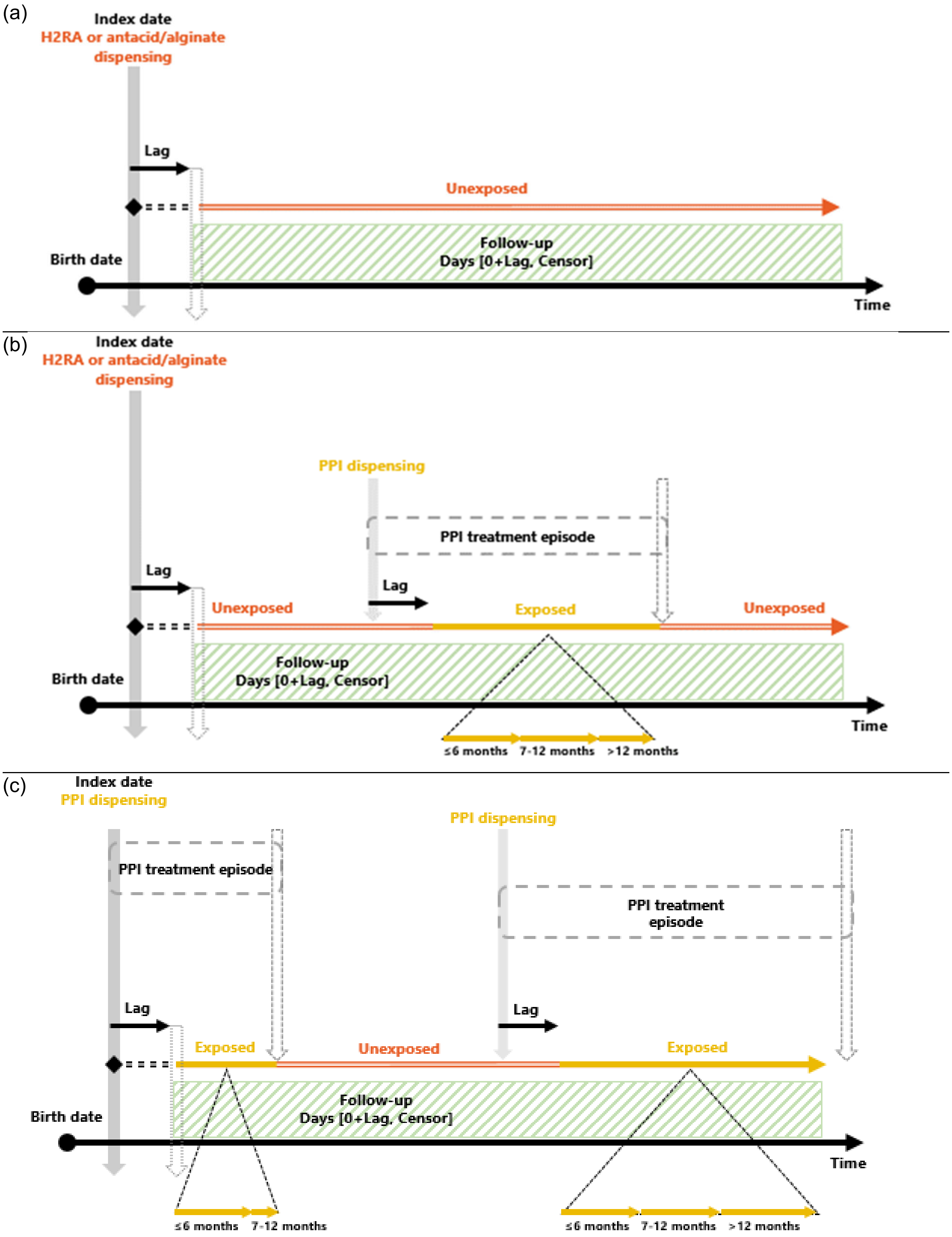

eFigure 3. PPI use over time defined as time-varying according to duration of any ongoing exposure – Examples

© 2023 Lassalle M et al. *JAMA Pediatrics.*

eFigure 3

Index date: first dispensing of a drug treatment for reflux or other gastric acid-related disorders, namely proton pump inhibitor (PPI), histamine-2-receptor antagonist ([H2RA], or antacid/alginate.

PPI treatment episode: one or several PPI dispensings, with (at most) 90-day gaps between two consecutive dispensings.

Follow-up: Children were followed from the index date (day 0)+lag until occurrence of serious infection, loss to follow-up (censored 1 year after the last identified date of outpatient care), death, or December 31, 2019, whichever came first.

(a) In this example, the child was dispensed an H2RA or antacid/alginate at index date, and did not receive any PPI dispensing during follow-up. Thus, she/he exclusively contributed as unexposed person-time.

(b) In this example, the child was dispensed an H2RA or antacid/alginate at index date, then dispensed PPI in a single PPI treatment episode lasting above 12 months during follow-up. Thus, she/he contributed successively as: unexposed person-time (from the start of the follow-up to the start of PPI treatment episode+lag), ≤6 months exposed person-time (from the start of PPI treatment episode+lag to the end of the 6th month of PPI treatment episode), 7-12 months exposed person-time (from the end of the 6th month of PPI treatment episode to the end of the 12th month of the end of PPI treatment episode), >12 months exposed person-time (from the end of the 12th month of PPI treatment episode to the end of PPI treatment episode), then unexposed person-time (from the end of PPI treatment episode to the end of follow-up).

(c) In this example, the child was dispensed a PPI at index date. In total, during follow-up, he/she received two PPI treatment episodes, the first one lasting above 6 months and less than 12 months, the second one lasting more than 12 months. Thus, she/he contributed successively as ≤6 months exposed person-time, unexposed person-time, ≤6 months exposed person-time, 7-12 months exposed person-time, then >12 months exposed person-time until the end of follow-up.

© 2023 Lassalle M et al. *JAMA Pediatrics.*

eTables

eTable 1. List of ICD-10 codes used to identify serious infections

Infection site	
**Digestive tract**	
Gastrointestinal and oesophageal infections	A00–A08, A090, K2380, K93820
Viral hepatitis	B15–B19, B251
Liver abscess	K750
Cholangitis	K800, K801, K803, K804, K810, K830, K8700
**ENT sphere**	
Mastoiditis	H70, H750
Nasopharyngitis	A361
Rhinitis	J00
Sinusitis	J01
Pharyngitis	J02
Pharyngeal, retropharyngeal, and parapharyngeal abscess	J36, J390, J391
Tonsillitis	A360, J03
Laryngitis and epiglottitis	A362, J05
Acute upper respiratory infections of multiple and unspecified sites	A368, A369, J06
Infection of external ear and acute otitis media	H600–H603, H620–H623, H651, H66, H670, H671, H680
**Lower respiratory tract**	
Pneumonia	A481, B012, B052, B250, J09–J18
Lung abscess	J85
Pleural empyema	J86
Other acute lower respiratory infections	A37, A420, B39, B40, B44, B583, B59, B953, J20–J22, U04
**Kidneys or urinary tract**	
Acute pyelonephritis	N10, N136
Acute cystitis	N300
Urinary tract infection of unspecified site	N390
**Skin**	
Erysipelas	A46
Dermatophytosis and other superficial mycoses	B35, B36, B372, B379
Cellulitis and abscess	J340, L02, L03
Other local infections of skin, oral tissue, and subcutaneous tissue	L00, L01, L04, L05, L08, L303, M726
**Musculoskeletal system**	M00, M01, M463, M462, M465, M491–M493, M600, M630–M632, M650, M651, M86, M901, M902
**Nervous system**	
Viral meningitis	A87, B003, B010, B021, B051, G020
Bacterial meningitis	A321, A39, G00, G01
Encephalitis and other neurological infections	A80–A89, B004, B011, B020, B022, B050, B060, G021, G028, G04–G07
**Others**	
Eye infections	B005, B30, B580, H000, H010, H030–H031, H061, H105,

© 2023 Lassalle M et al. *JAMA Pediatrics.*

	H130, H131, H191, H192, H220
Infections of prosthetic devices, implants, and grafts	T826, T827, T845–T847, T857
Acute infective pericarditis, myocarditis, and endocarditis	I301, I320, I321, I330, I400, I410–I412, I520, I521
Sepsis, systemic inflammatory response syndrome (SIRS) of infectious origin and septic shock	A327, A40, A41, R572, R650, R651
Unspecified infectious diseases	B99
Infectious pathogens	
**Bacterial**	A00–A05, A403, A20–A28, A32–A40, A410–A415, A42–A57, A65–A79, B95-B97, G00, G01, G042, G050, H000, H010, H105, H600–H603, H620, H651, H66, H670, H70, H750, I320, I410, I520, I980, J020, J030, J051, J13–J15, J160, J170, J200–J202, J340, J36, J390–J391, K113, K122, K670–K672, K800, K801, K803–K804, K810, K830, L00–L03, L05, L080,L081, M00, M010, M013, M491, M492, M630, M726, N136, N300, N410, N412, N413
**Viral**	A08, A60, A80–A99, B00, B02, B05, B06, B08, B09, B15-B27, B30, B33–B34, G020, G041, G051, H191, H621, H671, I411, J050, J09–J12, J171, J203–J207, J21, K2380, K8700, K93820, M014, M015
**Others**	B35–B49, B59, G021, H622, J172, M016<br>A06, A07, A59, B50–B58, B60, B64–B83, B87, H030, H061, H130, J173, M012, M631<br>A15–A19, A30, A31, K230, K673, K930, M011, M490, M900, N330, N740, N741<br>A090, A418–A419, B99, G028, G040, G048–G049, G052–G058, G06–G07, H031, H131, H192, H220, H623, H680, I301, I321, I330, I400, I412, I521, J00–J01, J028–J029, J038–J039, J06, J168, J178, J18, J208–J209, J22, J85–J86, K750, L04, L088–L089, L303, M018, M462–M463, M465, M493, M600, M632, M650–M651, M86, M901–M902, N10, N390, N700, N710, N72, N733, N771, P378-P379, P39, R572, R650–R651, T826–T827, T845–T847, T857, U04

© 2023 Lassalle M et al. *JAMA Pediatrics.*

eTable 2. List of codes used to identify maternal comorbidities

Diabetes	
ICD codes	E10-E14, O24
ATC codes	A10 (excluding A10BX06)
Obesity	
ICD codes	E66
Hypertension	
ICD codes	I10-I13, I15, O10-O13, O14-O16
ATC codes	C02AB02, C02AC01, C02AC02, C02AC05, C02AC06, C02CA01, C02CA06, C02DC01, C02LA01, C03AA01, C03AA03, C03BA04, C03BA10, C03BA11, C03BX03, C03CA01, C03CA02, C03CA03, C03DA01, C03DB01, C03EA, C03EA01, C03EA04, C07AA02, C07AA03, C07AA05, C07AA06, C07AA12, C07AA15, C07AA16, C07AA23, C07AB02, C07AB03, C07AB04, C07AB05, C07AB07, C07AB08, C07AB12, C07AG01, C07BA02, C07BB02, C07BB03, C07BB07, C07BB12, C07CA03, C07DA06, C07FB02, C07FB03, C08CA01, C08CA02, C08CA03, C08CA04, C08CA05, C08CA08, C08CA09, C08CA11, C08CA13, C08CX01, C08DA01, C08DB01, C08GA02, C09AA01, C09AA02, C09AA03, C09AA04, C09AA05, C09AA06, C09AA07, C09AA08, C09AA09, C09AA10, C09AA13, C09AA15, C09AA16, C09BA01, C09BA02, C09BA03, C09BA04, C09BA05, C09BA06, C09BA07, C09BA09, C09BA15, C09BB02, C09BB04, C09BB10, C09BX02, C09CA01, C09CA02, C09CA03, C09CA04, C09CA06, C09CA07, C09CA08, C09DA01, C09DA02, C09DA03, C09DA04, C09DA06, C09DA07, C09DA08, C09DB01, C09DB02, C09DB04, C09XA02, C09XA52, C10BX03
Alcohol consumption	
ICD codes	E244, F10, G312, G621, G721, I426, K292, K70, K852, K860, R780, T506, T510, X45, X65, Y15, Y573, Y90, Y91, Z502, Z714, Z721
ATC codes	N07BB
Tobacco consumption	
ICD codes	F17, T652, Z587, Z716, Z720, J41-J44
ATC codes	N07BA, N06AX12
Illicit substance consumption	
ICD codes	F11-F16, F18, F19, R781-R785, T40, X62, Y12, Z722
ATC codes	N07BC01, N07BC02, N07BC51

© 2023 Lassalle M et al. *JAMA Pediatrics.*

eTable 3. List of codes used to identify child comorbidities and drug exposures

Chronic respiratory diseases including major congenital airway malformations	
ICD codes	J40-J47, J961, J98, E84, Q300, Q321, Q323, Q324, Q330, Q332, Q333, Q334, Q335, Q336, Q338, Q339, Q34
ATC codes	R03
Neurological or degenerative diseases including major congenital malformations of the nervous system	
ICD codes	G35, G82, G70-G73, G40, G41, Q04-Q07
ATC codes	L03AB07, L03AB08, L03AB13, L03AX13, L04AA23, L04AA27, L04AA31, N07XX07, N07XX09
Diabetes	
ICD codes	E10-E14
ATC codes	A10 (excluding A10BX06)
Obesity	
ICD codes	E66
Liver diseases including major congenital liver malformations	
ICD codes	K70-K76, Q446, Q447
Chronic kidney diseases and major congenital malformations of the urinary system	
ICD codes	N18, Z992, Q60, Q611-Q615, Q618-Q626, Q628, Q630-Q632, Q638, Q639, Q64
CCAM codes	JVJB001, JVJF004, JVJF008, JVRP004, JVRP007, JVRP008, YYYY007
Cardiovascular diseases including major congenital heart defects	
ICD codes	I01, I05-I09, I11, I13, I20-I25, I27, I30-I45, I47-I52, I65, I66, I71, I72, I77-I83, I87, I95, I99, Z994, Q20-Q25, Q260, Q262-Q266, Q268, Q269
Indications for PPI	
ICD codes	
Autoimmune diseases or other sources of potential immunosuppression and major congenital chromosomal defects	
ICD codes	Cancers: C00-C97, D00-D09, Z510, Z511<br>MICI: K50, K51
	Rheumatoid arthritis and related diseases: M05, M06, M08 (excluding M081), M09
	Ankylosing spondylitis and related diseases: M07 (excluding M074, M075), M081, M45, M46
	Other chronic inflammatory diseases: L93, L94, M30-M36
	Inherited metabolic disorders or amyloidosis:<br>Hospital diagnosis: D510-D512, D552, D740, E071, E250, E700-E703, E710, E711, E713, E720-E725, E728, E730, E740-E744, E748, E750-E752, E754, E755, E760, E761, E762, E770, E771, E778, E786, E800, E801, E803, E805, E806, E830-E832, G601, Q773, Q871

© 2023 Lassalle M et al. *JAMA Pediatrics.*

	LTD: E85, D51, D53, D55, D74, E07, E13, E20, E25, E53, E56, E58, E70-E79, E80, E83, E88, G60, G71, H47, H49, Q77, Q87
	Haemophilia:<br>Hospital diagnosis: D66, D67<br>LTD: D66-D69
	Psoriasis: L40
	Sarcoidosis: D86
	Dermatomyositis: M608, M609, G724
	Relapsing polychondritis: M941
	Interstitial and deep keratitis: H163, H518
	Certain disorders involving the immune mechanism: D80-D84, D86, D89
	Asplenia/ Hyposplenism: Q890, D730
	Q90-Q92, Q930-Q935, Q937-Q939, Q96-Q99
ATC codes	Immunosuppressants: L04<br>Antipsoriatics for topical use: D05AX02, D05AX03, D05AX04, D05AX05, D05AX52
CCAM codes	Transplantation: DZEA001, DZEA002, DZEA003, DZEA004, HLEA001, HLEA002, GFEA001, GFEA002, GFEA003, GFEA004, GFEA005, GFEA006, GFEA007, HGEA002, HGEA004, HGEA005, HNEA002, HNEA900, JAEA003<br>Splenectomy: FFFA001, FFFA002, FFFC001, FFFC420
Digestive diseases including congenital malformations of the digestive system	
ICD codes	B980, E164, K20-K23, K25-K28-K31, K44, K92, R101, R12, P920, P921, P928, P929, F982, Q380, Q383-Q388, Q39, Q40-Q45, Q790
Corticosteroids	
ATC codes	H02AB
NSAID	
ATC codes	M01A

CCAM: French coding system of surgical procedures (“Classification Commune des Actes Médicaux”)
LTD: long-term disease

© 2023 Lassalle M et al. *JAMA Pediatrics.*

eTable 4. List of ICD-10 codes used to identify traumatic injuries, excluding fractures

Traumatic injury	
ICD codes	V01-V99, W00-W99, X00-X59
Fractures (excluded)	
ICD codes	S02, S12, S22, S32, S42, S52, S62, S72, S82, S92, T02, T08, T10, T12, T142

© 2023 Lassalle M et al. *JAMA Pediatrics.*

eTable 5. Description of the study population at baseline

	All (n=1,262,424)	Received PPIs during follow-up (n=606,645)	Did not receive PPIs during follow-up (n=655,779)	ASD
Sociodemographic characteristics, No. (%)				
Age at index date, median [IQR], days	84 (44-210)	88 (44-282)	82 (44-172)	0.224
Male	666,306 (52.8)	323,852 (53.4)	342,454 (52.2)	0.023
CMUC	115,583 (9.2)	46,325 (7.6)	69,258 (10.6)	-0.102
Deprivation index				0.230
1 (least deprived)	279,614 (22.2)	162,061 (26.7)	117,553 (17.9)	
2	256,927 (20.4)	124,780 (20.6)	132,147 (20.2)	
3	234,426 (18.6)	104,563 (17.2)	129,863 (19.8)	
4	221,451 (17.5)	97,812 (16.1)	123,639 (18.9)	
5 (most deprived)	231,219 (18.3)	99,107 (16.3)	132,112 (20.2)	
Missing	38,787 (3.1)	18,322 (3.0)	20,465 (3.1)	
Urban unit				0.142
≥200,000 inhabitants	598,855 (47.4)	310,067 (51.1)	288,788 (44.0)	
50,000-199,999 inhabitants	153,888 (12.2)	69,943 (11.5)	83,945 (12.8)	
10,000-49,999 inhabitants	121,914 (9.7)	55,375 (9.1)	66,539 (10.2)	
2,000 à 9,999 inhabitants	140,659 (11.1)	62,930 (10.4)	77,729 (11.9)	
Outside urban unit	219,151 (17.4)	96,245 (15.9)	122,906 (18.7)	
Missing	27,957 (2.2)	12,085 (2.0)	15,872 (2.4)	
Pregnancy and delivery characteristics, No. (%)				
Maternal age at start of pregnancy				
Median [IQR]	29 (26-33)	30 (26-33)	29 (26-33)	0.104
≥35 years old	204,442 (16.2)	103,341 (17.0)	101,101 (15.4)	0.044
Assisted reproductive technology	43,923 (3.5)	23,119 (3.8)	20,804 (3.2)	0.035
Maternity status				0.041
Public	809,006 (64.1)	382,494 (63.1)	426,512 (65.0)	
Private	452,615 (35.9)	223,750 (36.9)	228,865 (34.9)	
Missing	803 (0.1)	401 (0.1)	402 (0.1)	
Mode of delivery				0.037
Vaginal	997,980 (79.1)	474,852 (78.3)	523,128 (79.8)	
Caesarean	263,479 (20.9)	131,324 (21.6)	132,155 (20.2)	
Missing	965 (0.1)	469 (0.1)	496 (0.1)	
Gestational age				0.058
Full-term	1,175,831 (93.1)	560,990 (92.5)	614,841 (93.8)	
Preterm:	86,593 (6.9)	45 655 (7.5)	40,938 (6.2)	
Moderate to late preterm	78,099 (6.2)	40,513 (6.7)	37,586 (5.7)	

© 2023 Lassalle M et al. *JAMA Pediatrics.*

	All (n=1,262,424)	Received PPIs during follow-up (n=606,645)	Did not receive PPIs during follow-up (n=655,779)	ASD
Very preterm	7,181 (0.6)	4,302 (0.7)	2,879 (0.4)	
Extremely preterm	1,313 (0.1)	840 (0.1)	473 (0.1)	
Birth weight				0.018
Severe macrosomia	53,340 (4.2)	25,692 (4.2)	27,648 (4.2)	
Macrosomia	79,541 (6.3)	38,025 (6.3)	41,516 (6.3)	
Normal weight	941,279 (74.6)	450,899 (74.3)	490,380 (74.8)	
Small weight	94,769 (7.5)	45,740 (7.5)	49,029 (7.5)	
Severe low weight	51,576 (4.1)	25,823 (4.3)	25,753 (3.9)	
Missing	41 919 (3.3)	20,466 (3.4)	21,453 (3.3)	
Maternal comorbidities, No. (%)				
Diabetes	118,910 (9.4)	57,398 (9.5)	61,512 (9.4)	0.003
Hypertension	71,242 (5.6)	36,502 (6.0)	34,740 (5.3)	0.031
Obesity	67,802 (5.4)	30,698 (5.1)	37,104 (5.7)	-0.027
Consumption of tobacco	63,999 (5.1)	27,874 (4.6)	36,125 (5.5)	-0.042
Consumption of alcohol	920 (0.1)	419 (0.1)	501 (0.1)	-0.003
Consumption of illicit substances	3,782 (0.3)	1,614 (0.3)	2,168 (0.3)	-0.012
Child comorbidities and drug exposures at index date, No. (%)				
Respiratory diseases	72,608 (5.8)	48,972 (8.1)	23,636 (3.6)	0.191
Neurological diseases	3,870 (0.3)	2,705 (0.5)	1,165 (0.2)	0.048
Diabetes	133 (0.0)	80 (0.0)	53 (0.0)	0.005
Obesity	127 (0.0)	86 (0.0)	41 (0.0)	0.008
Liver diseases	477 (0.0)	345 (0.1)	132 (0.0)	0.019
Chronic kidney diseases	5,452 (0.4)	2,724 (0.5)	2,728 (0.4)	0.005
Cardiovascular diseases	12,726 (1.0)	7,678 (1.3)	5,048 (0.8)	0.049
Immunosuppression	6,081 (0.5)	4,599 (0.8)	1,482 (0.2)	0.076
Digestive diseases	63,227 (5.0)	39,497 (6.5)	23,730 (3.6)	0.132
Chronic corticosteroid treatment	59,424 (4.7)	40,251 (6.6)	19,173 (2.9)	0.175
NSAID treatment	44,313 (3.5)	28,034 (4.6)	16,279 (2.5)	0.198
Healthcare use, No. (%)				
In mothers				
≥3 preventive drugs dispensed during pregnancy	769,941 (61.0)	374,016 (61.7)	395,925 (60.4)	0.026
In children, within 3 months before index date				
≥2 outpatient visits, all medical specialties	947,024 (75.0)	454,960 (75.0)	492,064 (75.0)	0.001
≥2 pediatric outpatient visits	417,040 (33.0)	216,583 (35.7)	200,457 (30.6)	0.109
≥2 drug dispensings	740,464 (58.7)	355,287 (58.6)	385,177 (58.7)	0.004
≥1 hospital stays	82,723 (6.6)	51,581 (8.5)	31,142 (4.8)	0.151

Abbreviations: ASD, absolute standardized differences; CMUC, complementary universal health insurance; NSAID, nonsteroidal anti-inflammatory drug; PPI, proton pump inhibitor.

© 2023 Lassalle M et al. *JAMA Pediatrics.*

eTable 6. Overall risk of serious infections associated with covariates

	Crude HR (95% CI)	aHR[a] (95% CI)
Serious infections, overall		
**Sociodemographic characteristics**		
Age at index date		
<1 year	1 (Ref.)	1 (Ref.)
≥1 year	1.14 (1.11-1.17)	1.07 (1.05-1.10)
Sex		
Females	1 (Ref.)	1 (Ref.)
Males	1.06 (1.05-1.07)	1.03 (1.02-1.04)
CMUC		
No	1 (Ref.)	1 (Ref.)
Yes	1.38 (1.36-1.40)	1.22 (1.20-1.24)
Deprivation index		
1 (least deprived)	1 (Ref.)	1 (Ref.)
2	1.17 (1.15-1.19)	1.10 (1.08-1.12)
3	1.31 (1.29-1.33)	1.17 (1.15-1.19)
4	1.42 (1.39-1.44)	1.21 (1.19-1.23)
5 (most deprived)	1.60 (1.57-1.62)	1.33 (1.30-1.35)
Urban unit		
≥200,000 inhabitants	1 (Ref.)	1 (Ref.)
50,000-199,999 inhabitants	1.31 (1.29-1.33)	1.15 (1.14-1.17)
10,000-49,999 inhabitants	1.44 (1.42-1.46)	1.24 (1.22-1.27)
2,000 à 9,999 inhabitants	1.17 (1.15-1.19)	1.06 (1.04-1.08)
Outside urban unit	1.20 (1.06-1.14)	1.09 (1.08-1.11)
**Pregnancy and delivery characteristics**		
Maternal age at start of pregnancy		
<35 years	1 (Ref.)	1 (Ref.)
≥35 years	0.85 (0.83-0.86)	0.86 (0.85-0.87)
Assisted reproductive technology		
No	1 (Ref.)	1 (Ref.)
Yes	0.98 (0.95-1.01)	1.01 (0.98-1.03)
Maternity status		
Public	1 (Ref.)	1 (Ref.)
Private	0.75 (0.74-0.76)	0.84 (0.83-0.85)
Mode of delivery		

© 2023 Lassalle M et al. *JAMA Pediatrics.*

	Crude HR (95% CI)	aHR[a] (95% CI)
Vaginal		1 (Ref.)
Caesarean	1.13 (1.12-1.15)	1.07 (1.06-1.09)
Gestational age		
Full-term	1 (Ref.)	1 (Ref.)
Moderate to late preterm	1.34 (1.31-1.36)	1.17 (1.15-1.19)
Very preterm	2.04 (1.95-2.15)	1.37 (1.30-1.44)
Extremely preterm	3.10 (2.80-3.43)	1.92 (1.73-2.13)
Birth weight		
Normal weight	1 (Ref.)	1 (Ref.)
Severe macrosomia	0.96 (0.94-0.98)	0.95 (0.93-0.98)
Macrosomia	1.00 (0.98-1.03)	0.95 (0.93-0.97)
Small weight	1.12 (1.10-1.14)	1.07 (1.05-1.09)
Severe low weight	1.31 (1.28-1.34)	1.11 (1.09-1.14)
**Maternal comorbidities**		
Diabetes		
No	1 (Ref.)	1 (Ref.)
Yes	1.07 (1.05-1.09)	1.02 (1.00-1.04)
Hypertension		
No	1 (Ref.)	1 (Ref.)
Yes	1.23 (1.21-1.25)	1.10 (1.08-1.12)
Obesity		
No	1 (Ref.)	1 (Ref.)
Yes	1.25 (1.23-1.28)	1.10 (1.07-1.12)
Tobacco consumption		
No	1 (Ref.)	1 (Ref.)
Yes	1.25 (1.23-1.28)	1.09 (1.07-1.11)
Alcohol consumption		
No	1 (Ref.)	1 (Ref.)
Yes	1.33 (1.13-1.57)	1.04 (0.88-1.23)
Illicit substance consumption		
No	1 (Ref.)	1 (Ref.)
Yes	1.15 (1.05-1.26)	0.88 (0.81-0.97)
**Child comorbidities and drug exposures over time**		
Respiratory diseases		
No	1 (Ref.)	1 (Ref.)
Yes	1.44 (1.42-1.46)	1.26 (1.24-1.28)

© 2023 Lassalle M et al. *JAMA Pediatrics.*

	Crude HR (95% CI)	aHR[a] (95% CI)
Neurological diseases		
No	1 (Ref.)	1 (Ref.)
Yes	2.79 (2.67-2.92)	1.86 (1.77-1.94)
Diabetes		
No	1 (Ref.)	1 (Ref.)
Yes	3.29 (2.71-3.99)	2.68 (2.21-3.25)
Obesity		
No	1 (Ref.)	1 (Ref.)
Yes	2.52 (1.95-3.27)	0.72 (0.56-0.94)
Liver diseases		
No	1 (Ref.)	1 (Ref.)
Yes	3.68 (3.26-4.15)	1.73 (1.53-1.95)
Chronic kidney diseases		
No	1 (Ref.)	1 (Ref.)
Yes	2.05 (1.94-2.16)	1.72 (1.63-1.81)
Cardiovascular diseases		
No	1 (Ref.)	1 (Ref.)
Yes	2.19 (2.11-2.26)	1.40 (1.35-1.45)
Immunosuppression		
No	1 (Ref.)	1 (Ref.)
Yes	3.57 (3.44-3.71)	2.46 (2.36-2.56)
Digestive diseases		
No	1 (Ref.)	1 (Ref.)
Yes	1.65 (1.62-1.67)	1.27 (1.25-1.29)
Chronic corticosteroid treatment		
No	1 (Ref.)	1 (Ref.)
Yes	1.42 (1.40-1.45)	1.21 (1.19-1.23)
NSAID treatment		
No	1 (Ref.)	1 (Ref.)
Yes	1.48 (1.46-1.49)	1.40 (1.38-1.41)
**Healthcare use**		
In mothers		
Number of dispensings of preventive drugs during pregnancy		
<3	1 (Ref.)	1 (Ref.)
≥3	0.99 (0.98-1.00)	1.01 (1.00-1.02)

© 2023 Lassalle M et al. *JAMA Pediatrics.*

	Crude HR (95% CI)	aHR[a] (95% CI)
In children, within 3 months before index date		
Number of outpatient visits, all medical specialties		
<2	1 (Ref.)	1 (Ref.)
≥2	0.92 (0.91-0.93)	0.98 (0.97-0.99)
Number of pediatric outpatient visits		
<2	1 (Ref.)	1 (Ref.)
≥2	0.86 (0.85-0.87)	0.97 (0.96-0.98)
Number of drug dispensings		
<2	1 (Ref.)	1 (Ref.)
≥2	1.04 (1.03-1.05)	1.04 (1.03-1.05)
Number of hospital stays		
<1	1 (Ref.)	1 (Ref.)
≥1	1.64 (1.61-1.66)	1.21 (1.18-1.23)
**Season over time**		
Spring	1 (Ref.)	1 (Ref.)
Summer	0.55 (0.54-0.56)	0.55 (0.54-0.56)
Fall	0.71 (0.70-0.72)	0.73 (0.72-0.75)
Winter	1.16 (1.15-1.18)	1.26 (1.24-1.28)
**Calendar year at index date**		
2010	1 (Ref.)	1 (Ref.)
2011	1.01 (0.99-1.03)	1.01 (0.99-1.03)
2012	1.01 (0.99-1.03)	1.01 (0.99-1.03)
2013	1.00 (0.98-1.02)	1.00 (0.98-1.02)
2014	1.03 (1.01-1.05)	1.03 (1.01-1.06)
2015	1.04 (1.01-1.06)	1.04 (1.02-1.07)
2016	1.03 (1.00-1.05)	1.04 (1.02-1.07)
2017	1.01 (0.98-1.03)	1.03 (1.01-1.06)
2018	1.03 (1.00-1.05)	1.06 (1.04-1.09)
2019	0.76 (0.73-0.82)	0.85 (0.81-0.90)

[a]Cox models adjusted for time-fixed covariates, namely sociodemographic characteristics: age at index date, sex, CMUC, deprivation index, size of the urban unit, calendar year at index date; pregnancy and delivery characteristics: maternal age, ART, maternity status, mode of delivery, gestational age, birth weight; maternal comorbidities: diabetes, hypertension, obesity, tobacco, alcohol, and illicit substance consumption; healthcare use in mothers: preventive medications during pregnancy; healthcare use in children: outpatient physician visits, outpatient pediatrician visits, drug dispensings, hospital stays; and for time-varying covariates, namely season, child comorbidities and drug exposures: respiratory diseases, neurological disease, diabetes, obesity, liver diseases, chronic kidney diseases, cardiovascular diseases, immunosuppression, digestive diseases, chronic corticosteroid treatment, NSAID treatment. For each categorical covariate, missing values, if any, were considered as a separate category.

© 2023 Lassalle M et al. *JAMA Pediatrics.*

eTable 7. Overall risk of serious infections associated with history of PPI exposure over time in children, distinguishing past PPI users according to time since PPI treatment withdrawal

	No. of events/No. of PY	Incidence rate[a] (95% CI)	Crude HR (95% CI)	aHR[b] (95% CI)
Serious infections, overall				
History of PPI exposure over time				
None	82,545/2,853,971	2.89 (2.87-2.91)	1 (Ref.)	1 (Ref.)
Past exposure, withdrawal since >12 months	19,027/1,387,163	1.37 (1.35-1.39)	1.04 (1.02-1.06)	1.03 (1.01-1.05)
Past exposure, withdrawal since 7-12 months	9,823/264,222	3.72 (3.64-3.79)	1.14 (1.11-1.16)	1.09 (1.07-1.11)
Past exposure, withdrawal since 4-6 months	6,718/145,666	4.61 (4.5-4.72)	1.12 (1.09-1.15)	1.08 (1.06-1.11)
Past exposure, withdrawal since ≤3 months	8,751/159,724	5.48 (5.37-5.59)	1.16 (1.13-1.18)	1.13 (1.10-1.16)
Ongoing	25,191/271,874	9.27 (9.15-9.38)	1.46 (1.43-1.48)	1.37 (1.35-1.39)

[a]Per 100 person-years (PY)
[b]Cox models adjusted for time-fixed covariates, namely sociodemographic characteristics: age at index date, sex, CMUC, deprivation index, size of the urban unit, calendar year at index date; pregnancy and delivery characteristics: maternal age, ART, maternity status, mode of delivery, gestational age, birth weight; maternal comorbidities: diabetes, hypertension, obesity, tobacco, alcohol, and illicit substance consumption; healthcare use in mothers: preventive medications during pregnancy; healthcare use in children: outpatient physician visits, outpatient pediatrician visits, drug dispensings, hospital stays; and for time-varying covariates, namely season, child comorbidities and drug exposures: respiratory diseases, neurological disease, diabetes, obesity, liver diseases, chronic kidney diseases, cardiovascular diseases, immunosuppression, digestive diseases, chronic corticosteroid treatment, NSAID treatment. For each categorical covariate, missing values, if any, were considered as a separate category.

© 2023 Lassalle M et al. *JAMA Pediatrics.*

eTable 8. Overall risk of serious infections associated with proton pump inhibitor exposure over time in children, separately in children born very/extremely preterm or with a chronic comorbidity[a], and in those without any of these conditions at baseline

	No. of events/No. of PY	Incidence rates[b] (95% CI)	Crude HR (95% CI)	aHR[c] (95% CI)
Serious infections, overall				
Children with no history of severe prematurity or chronic condition at baseline				
Unexposed	110,160/4,225,569	2.61 (2.59-2.62)	1 (Ref.)	1 (Ref.)
Exposed	19,654/220,673	8.90 (8.78-9.03)	1.35 (1.33-1.37)	1.32 (1.30-1.34)
Children born very or extremely preterm or with a chronic comorbidity[a] at baseline				
Unexposed	16,704/585,177	2.85 (2.81-2.90)	1 (Ref.)	1 (Ref.)
Exposed	5,537/51,200	10.81 (10.53-11.10)	1.39 (1.35-1.44)	1.36 (1.32-1.41)

[a]Chronic comorbidities or drug exposure identified at baseline: chronic respiratory diseases, neurological or degenerative diseases, diabetes, obesity, liver diseases, chronic kidney diseases and major congenital malformations of the urinary system, cardiovascular diseases, autoimmune diseases or other sources of potential immunosuppression, digestive diseases, and chronic corticosteroid treatment

[b]Per 100 person-years (PY)

[c]Cox models adjusted for time-fixed covariates, namely sociodemographic characteristics: age at index date, sex, CMUC, deprivation index, size of the urban unit, calendar year at index date; pregnancy and delivery characteristics: maternal age, ART, maternity status, mode of delivery, gestational age, birth weight; maternal comorbidities: diabetes, hypertension, obesity, tobacco, alcohol, and illicit substance consumption; healthcare use in mothers: preventive medications during pregnancy; healthcare use in children: outpatient physician visits, outpatient pediatrician visits, drug dispensings, hospital stays; and for time-varying covariates, namely season, child comorbidities and drug exposures: respiratory diseases, neurological disease, diabetes, obesity, liver diseases, chronic kidney diseases, cardiovascular diseases, immunosuppression, digestive diseases, chronic corticosteroid treatment, NSAID treatment. For each categorical covariate, missing values, if any, were considered as a separate category.

© 2023 Lassalle M et al. *JAMA Pediatrics.*

eTable 9. Overall risk of serious infections associated with PPI exposure over time in children, excluding H2RA users at baseline

	No. of events/No. of PY	Incidence rate[a] (95% CI)	Crude HR (95% CI)	aHR[b] (95% CI)
Serious infections, overall				
PPI exposure				
Unexposed	123,791/4,689,249	2.64 (2.63-2.65)	1 (Ref.)	1 (Ref.)
Exposed	24,478/263,359	9.29 (9.18-9.41)	1.42 (1.39-1.44)	1.34 (1.31-1.36)

[a]Per 100 person-years (PY)
[b]Cox models adjusted for time-fixed covariates, namely sociodemographic characteristics: age at index date, sex, CMUC, deprivation index, size of the urban unit, calendar year at index date; pregnancy and delivery characteristics: maternal age, ART, maternity status, mode of delivery, gestational age, birth weight; maternal comorbidities: diabetes, hypertension, obesity, tobacco, alcohol, and illicit substance consumption; healthcare use in mothers: preventive medications during pregnancy; healthcare use in children: outpatient physician visits, outpatient pediatrician visits, drug dispensings, hospital stays; and for time-varying covariates, namely season, child comorbidities and drug exposures: respiratory diseases, neurological disease, diabetes, obesity, liver diseases, chronic kidney diseases, cardiovascular diseases, immunosuppression, digestive diseases, chronic corticosteroid treatment, NSAID treatment. For each categorical covariate, missing values, if any, were considered as a separate category.

© 2023 Lassalle M et al. *JAMA Pediatrics.*

eTable 10. Risk of serious infections associated with proton pump inhibitor exposure over time in children, overall, by site and pathogen – Assessment of protopathic bias

	No. of events/No. of PY	Incidence rates[a] (95% CI)	Crude HR (95% CI)	aHR[b] (95% CI)
Serious infections, overall				
Variation of the lag time				
0-day lag				
Unexposed	137,389/4,853,287	2.83 (2.82-2.85)	1 (Reference)	1 (Ref.)
Exposed	36,170/331,197	10.92 (10.81-11.03)	1.47 (1.45-1.49)	1.39 (1.37-1.40)
7-day lag				
Unexposed	133,717/4,843,289	2.76 (2.75-2.78)	1 (Ref.)	1 (Ref.)
Exposed	32,523/317,236	10.25 (10.14-10.36)	1.45 (1.43-1.47)	1.37 (1.35-1.38)
30-day lag				
Unexposed	126,864/4,810,746	2.64 (2.62-2.65)	1 (Ref.)	1 (Ref.)
Exposed	25,191/271,874	9.27 (9.15-9.38)	1.42 (1.40-1.44)	1.34 (1.32-1.36)
60-day lag				
Unexposed	120,411/4,768,740	2.53 (2.51-2.54)	1 (Ref.)	1 (Ref.)
Exposed	18,186/213,675	8.51 (8.39-8.64)	1.41 (1.38-1.43)	1.32 (1.30-1.35)
Exclusion of children with antibiotic dispensing within 3 months before index date				
Unexposed	111,784/4,071,547	2.75 (2.73-2.76)	1 (Ref.)	1 (Ref.)
Exposed	22,328/224,977	9.92 (9.80-10.06)	1.41 (1.39-1.43)	1.34 (1.32-1.36)

© 2023 Lassalle M et al. *JAMA Pediatrics.*

	No. of events/No. of PY	Incidence rates[a] (95% CI)	Crude HR (95% CI)	aHR[b] (95% CI)
**By infection site**				
Digestive tract				
Variation of the lag time				
0-day lag				
Unexposed	51,944/5,276,567	0.98 (0.98-0.99)	1 (Ref.)	1 (Ref.)
Exposed	12,722/353,863	3.60 (3.53-3.66)	1.77 (1.73-1.81)	1.67 (1.63-1.70)
7-day lag				
Unexposed	51,492/5,267,013	0.98 (0.97-0.99)	1 (Ref.)	1 (Ref.)
Exposed	11,539/339,421	3.40 (3.34-3.46)	1.69 (1.65-1.73)	1.59 (1.56-1.63)
30-day lag				
Unexposed	50,608/5,235,608	0.97 (0.96-0.98)	1 (Ref.)	1 (Ref.)
Exposed	9,412/292,237	3.22 (3.16-3.29)	1.61 (1.57-1.65)	1.52 (1.48-1.55)
60-day lag				
Unexposed	49,500/5,194,589	0.95 (0.94-0.96)	1 (Ref.)	1 (Ref.)
Exposed	7,337/231,267	3.17 (3.10-3.25)	1.58 (1.54-1.62)	1.49 (1.45-1.53)
Exclusion of children with antibiotic dispensing within 3 months before index date				
Unexposed	44,844/4,446,093	1.01 (1.0-1.02)	1 (Ref.)	1 (Ref.)
Exposed	8,269/243,174	3.40 (3.33-3.47)	1.62 (1.58-1.67)	1.54 (1.50-1.58)
ENT sphere				
Variation of the lag time				
0-day lag				
Unexposed	26,144/5,415,669	0.48 (0.48-0.49)	1 (Ref.)	1 (Ref.)
Exposed	5,059/361,071	1.40 (1.36-1.44)	1.62 (1.57-1.68)	1.50 (1.45-1.55)
7-day lag	,	,	,	,
Unexposed	25,796/5,406,251	0.48 (0.47-0.48)	1 (Ref.)	1 (Ref.)
Exposed	4,626/346,485	1.34 (1.30-1.37)	1.61 (1.55-1.66)	1.48 (1.43-1.53)
30-day lag				
Unexposed	25,052/5,375,283	0.47 (0.46-0.47)	1 (Ref.)	1 (Ref.)
Exposed	3,700/298,771	1.24 (1.20-1.28)	1.60 (1.54-1.66)	1.47 (1.41-1.52)
60-day lag				
Unexposed	24,505/5,334,794	0.46 (0.45-0.47)	1 (Ref.)	1 (Ref.)
Exposed	2,835/237,027	1.20 (1.15-1.24)	1.60 (1.54-1.67)	1.45 (1.39-1.52)
Exclusion of children with antibiotic dispensing within 3 months before index date				

© 2023 Lassalle M et al. *JAMA Pediatrics.*

	No. of events/No. of PY	Incidence rates[a] (95% CI)	Crude HR (95% CI)	aHR[b] (95% CI)
Unexposed	21,645/4,571,140	0.47 (0.47-0.48)	1 (Ref.)	1 (Ref.)
Exposed	3,086/249,070	1.24 (1.20-1.28)	1.57 (1.50-1.63)	1.43 (1.38-1.50)
Lower respiratory tract				
Variation of the lag time				
0-day lag				
Unexposed	42,737/5,300,957	0.81 (0.80-0.81)	1 (Ref.)	1 (Ref.)
Exposed	15,379/351,518	4.38 (4.31-4.44)	1.34 (1.32-1.37)	1.23 (1.20-1.25)
7-day lag				
Unexposed	40,552/5,291,397	0.77 (0.76-0.77)	1 (Ref.)	1 (Ref.)
Exposed	13,763/337,096	4.08 (4.02-4.15)	1.35 (1.32-1.38)	1.22 (1.20-1.25)
30-day lag				
Unexposed	36,607/5,260,133	0.70 (0.69-0.70)	1 (Ref.)	1 (Ref.)
Exposed	10,446/290,030	3.60 (3.53-3.67)	1.35 (1.32-1.39)	1.22 (1.19-1.25)
60-day lag				
Unexposed	32,905/5,219,529	0.63 (0.62-0.64)	1 (Ref.)	1 (Ref.)
Exposed	7,255/229,321	3.16 (3.09-3.24)	1.37 (1.33-1.40)	1.22 (1.19-1.25)
Exclusion of children with antibiotic dispensing within 3 months before index date				
Unexposed	33,038/4,462,550	0.74 (0.73-0.75)	1 (Ref.)	1 (Ref.)
Exposed	9,625/240,899	4.0 (3.92-4.08)	1.35 (1.32-1.39)	1.22 (1.19-1.25)
Kidneys or urinary tract				
Variation of the lag time				
0-day lag				
Unexposed	14,057/5,456,162	0.26 (0.25-0.26)	1 (Ref.)	1 (Ref.)
Exposed	3,932/363,101	1.08 (1.05-1.12)	1.26 (1.21-1.31)	1.22 (1.18-1.27)
7-day lag				
Unexposed	13,676/5,446,801	0.25 (0.25-0.26)	1 (Ref.)	1 (Ref.)
Exposed	3,610/348,457	1.04 (1.0-1.07)	1.26 (1.21-1.31)	1.22 (1.17-1.27)
30-day lag				
Unexposed	12,826/5,416,027	0.24 (0.23-0.24)	1 (Ref.)	1 (Ref.)
Exposed	2,798/300,543	0.93 (0.90-0.97)	1.23 (1.18-1.29)	1.20 (1.15-1.25)
60-day lag				
Unexposed	11,950/5,375,805	0.22 (0.22-0.23)	1 (Ref.)	1 (Ref.)
Exposed	1,936/238,536	0.81 (0.78-0.85)	1.19 (1.13-1.25)	1.16 (1.10-1.22)

© 2023 Lassalle M et al. *JAMA Pediatrics.*

	No. of events/No. of PY	Incidence rates[a] (95% CI)	Crude HR (95% CI)	aHR[b] (95% CI)
Exclusion of children with antibiotic dispensing within 3 months before index date				
Unexposed	11,462/4,602,952	0.25 (0.24-0.25)	1 (Ref.)	1 (Ref.)
Exposed	2,523/250,443	1.01 (0.97-1.05)	1.23 (1.17-1.28)	1.19 (1.14-1.25)
Skin				
Variation of the lag time				
0-day lag				
Unexposed	6,173/5,509,638	0.11 (0.11-0.11)	1 (Ref.)	1 (Ref.)
Exposed	458/366,239	0.13 (0.11-0.14)	1.20 (1.08-1.33)	1.13 (1.02-1.25)
7-day lag				
Unexposed	6,165/5,500,336	0.11 (0.11-0.11)	1 (Ref.)	1 (Ref.)
Exposed	420/351,533	0.12 (0.11-0.13)	1.15 (1.04-1.28)	1.08 (0.98-1.21)
30-day lag				
Unexposed	6,127/5,469,711	0.11 (0.11-0.11)	1 (Ref.)	1 (Ref.)
Exposed	360/303,384	0.12 (0.11-0.13)	1.16 (1.03-1.29)	1.08 (0.97-1.21)
60-day lag				
Unexposed	6,113/5,429,612	0.11 (0.11-0.12)	1 (Ref.)	1 (Ref.)
Exposed	285/241,009	0.12 (0.11-0.13)	1.13 (1.0-1.28)	1.05 (0.92-1.19)
Exclusion of children with antibiotic dispensing within 3 months before index date				
Unexposed	5,109/4,652,561	0.11 (0.11-0.11)	1 (Ref.)	1 (Ref.)
Exposed	290/253,053	0.11 (0.10-0.13)	1.18 (1.04-1.33)	1.10 (0.97-1.25)
Musculoskeletal system				
Variation of the lag time				
0-day lag				
Unexposed	2,456/5,520,959	0.04 (0.04-0.05)	1 (Ref.)	1 (Ref.)
Exposed	256/366,458	0.07 (0.06-0.08)	1.54 (1.35-1.76)	1.32 (1.15-1.51)
7-day lag				
Unexposed	2,458/5,511,662	0.04 (0.04-0.05)	1 (Ref.)	1 (Ref.)
Exposed	239/351,747	0.07 (0.06-0.08)	1.48 (1.29-1.70)	1.27 (1.10-1.46)
30-day lag				
Unexposed	2,473/5,481,052	0.05 (0.04-0.05)	1 (Ref.)	1 (Ref.)
Exposed	203/303,579	0.07 (0.06-0.08)	1.38 (1.19-1.60)	1.17 (1.01-1.37)
60-day lag				

© 2023 Lassalle M et al. *JAMA Pediatrics.*

	No. of events/No. of PY	Incidence rates[a] (95% CI)	Crude HR (95% CI)	aHR[b] (95% CI)
Unexposed	2,480/5,440,967	0.05 (0.04-0.05)	1 (Ref.)	1 (Ref.)
Exposed	164/241,179	0.07 (0.06-0.08)	1.30 (1.11-1.53)	1.09 (0.93-1.29)
Exclusion of children with antibiotic dispensing within 3 months before index date				
Unexposed	2,147/4,661,842	0.05 (0.04-0.05)	1 (Ref.)	1 (Ref.)
Exposed	162/253,211	0.06 (0.05-0.07)	1.41 (1.19-1.66)	1.18 (0.99-1.39)
Nervous system				
Variation of the lag time				
0-day lag				
Unexposed	1,998/5,513,452	0.04 (0.03-0.04)	1 (Ref.)	1 (Ref.)
Exposed	293/351,607	0.08 (0.07-0.09)	1.59 (1.38-1.82)	1.44 (1.26-1.64)
7-day lag				
Unexposed	1,998/5,513,452	0.04 (0.03-0.04)	1 (Ref.)	1 (Ref.)
Exposed	293/351,607	0.08 (0.07-0.09)	1.59 (1.38-1.82)	1.40 (1.22-1.62)
30-day lag				
Unexposed	1,914/5,482,847	0.03 (0.03-0.04)	1 (Ref.)	1 (Ref.)
Exposed	200/303,443	0.07 (0.06-0.08)	1.50 (1.27-1.76)	1.31 (1.11-1.54)
60-day lag				
Unexposed	1,861/5,442,775	0.03 (0.03-0.04)	1 (Ref.)	1 (Ref.)
Exposed	136/241,051	0.06 (0.05-0.07)	1.50 (1.24-1.82)	1.28 (1.05-1.55)
Exclusion of children with antibiotic dispensing within 3 months before index date				
Unexposed	1,640/4,663,168	0.04 (0.03-0.04)	1 (Ref.)	1 (Ref.)
Exposed	170/253,084	0.07 (0.06-0.08)	1.44 (1.21-1.73)	1.26 (1.05-1.51)

© 2023 Lassalle M et al. *JAMA Pediatrics.*

	No. of events/No. of PY	Incidence rates[a] (95% CI)	Crude HR (95% CI)	aHR[b] (95% CI)
**By infection pathogen**				
Bacterial				
Variation of the lag time				
0-day lag				
Unexposed	25,126/5,426,890	0.46 (0.46-0.47)	1 (Ref.)	1 (Ref.)
Exposed	4,074/361,940	1.13 (1.09-1.16)	1.82 (1.76-1.89)	1.62 (1.56-1.68)
7-day lag				
Unexposed	24,943/5,417,493	0.46 (0.45-0.47)	1 (Ref.)	1 (Ref.)
Exposed	3,772/347,332	1.09 (1.05-1.12)	1.81 (1.74-1.87)	1.59 (1.53-1.66)
30-day lag				
Unexposed	24,715/5,386,573	0.46 (0.45-0.46)	1 (Ref.)	1 (Ref.)
Exposed	3,177/299,527	1.06 (1.02-1.10)	1.78 (1.71-1.85)	1.56 (1.50-1.63)
60-day lag				
Unexposed	24,565/5,346,109	0.46 (0.45-0.47)	1 (Ref.)	1 (Ref.)
Exposed	2,546/237,638	1.07 (1.03-1.11)	1.75 (1.68-1.83)	1.53 (1.46-1.60)
Exclusion of children with antibiotic dispensing within 3 months before index date				
Unexposed	21,007/4,582,788	0.46 (0.45-0.46)	1 (Ref.)	1 (Ref.)
Exposed	2,554/249,835	1.02 (0.98-1.06)	1.74 (1.67-1.82)	1.53 (1.47-1.60)
Viral				
Variation of the lag time				
0-day lag				
Unexposed	65,643/5,182,911	1.27 (1.26-1.28)	1 (Ref.)	1 (Ref.)
Exposed	21,203/346,244	6.12 (6.04-6.21)	1.42 (1.40-1.45)	1.35 (1.33-1.37)
7-day lag				
Unexposed	63,301/5,173,239	1.22 (1.21-1.23)	1 (Ref.)	1 (Ref.)
Exposed	19,002/331,939	5.72 (5.64-5.81)	1.40 (1.38-1.43)	1.33 (1.30-1.35)
30-day lag				
Unexposed	58,833/5,141,632	1.14 (1.14-1.15)	1 (Ref.)	1 (Ref.)
Exposed	14,598/285,310	5.12 (5.03-5.20)	1.38 (1.36-1.41)	1.30 (1.28-1.33)
60-day lag				
Unexposed	54,477/5,100,636	1.07 (1.06-1.08)	1 (Ref.)	1 (Ref.)
Exposed	10,414/225,248	4.62 (4.54-4.71)	1.38 (1.35-1.41)	1.30 (1.27-1.33)
Exclusion of children with antibiotic dispensing within 3 months before index date				

© 2023 Lassalle M et al. *JAMA Pediatrics.*

	No. of events/No. of PY	Incidence rates[a] (95% CI)	Crude HR (95% CI)	aHR[b] (95% CI)
Unexposed	52,910/4,356,888	1.21 (1.20-1.22)	1 (Ref.)	1 (Ref.)
Exposed	13,326/236,595	5.63 (5.54-5.73)	1.39 (1.36-1.42)	1.31 (1.28-1.34)

[a]Per 100 person-years (PY)

[b]Cox models adjusted for time-fixed covariates, namely sociodemographic characteristics: age at index date, sex, CMUC, deprivation index, size of the urban unit, calendar year at index date; pregnancy and delivery characteristics: maternal age, ART, maternity status, mode of delivery, gestational age, birth weight; maternal comorbidities: diabetes, hypertension, obesity, tobacco, alcohol, and illicit substance consumption; healthcare use in mothers: preventive medications during pregnancy; healthcare use in children: outpatient physician visits, outpatient pediatrician visits, drug dispensings, hospital stays; and for time-varying covariates, namely season, child comorbidities and drug exposures: respiratory diseases, neurological disease, diabetes, obesity, liver diseases, chronic kidney diseases, cardiovascular diseases, immunosuppression, digestive diseases, chronic corticosteroid treatment, NSAID treatment. For each categorical covariate, missing values, if any, were considered as a separate category.

© 2023 Lassalle M et al. *JAMA Pediatrics.*

eTable 11. Risk of serious lower respiratory tract infections in the 30 days before index date according to the treatment initiated at index date (PPI *vs.* H2RA or antacid/alginate)

	No. of events/No. of PY	Incidence rate[a] (95% CI)	Crude HR (95% CI)	aHR[b] (95% CI)
Lower respiratory tract infection				
PPI exposure at index date				
No	7,045/469,349	1.50 (1.47-1.54)	1 (Ref.)	1 (Ref.)
Yes	5,622/524,695	1.07 (1.04-1.10)	1.20 (1.16-1.24)	0.91 (0.87-0.94)

[a]Per 100 person-years (PY)
[b]Cox models adjusted for time-fixed covariates measured at baseline, namely sociodemographic characteristics: age at index date, sex, CMUC, deprivation index, size of the urban unit, calendar year at index date; pregnancy and delivery characteristics: maternal age, ART, maternity status, mode of delivery, gestational age, birth weight; maternal comorbidities: diabetes, hypertension, obesity, tobacco, alcohol, and illicit substance consumption; healthcare use in mothers: preventive medications during pregnancy; healthcare use in children: outpatient physician visits, outpatient pediatrician visits, drug dispensings, hospital stays; season; child comorbidities and drug exposures: respiratory diseases, neurological disease, diabetes, obesity, liver diseases, chronic kidney diseases, cardiovascular diseases, immunosuppression, digestive diseases, chronic corticosteroid treatment, NSAID treatment. For each categorical covariate, missing values, if any, were considered as a separate category.

© 2023 Lassalle M et al. *JAMA Pediatrics.*

eTable 12. E-values calculated for the association between PPI exposure over time and serious infections in children, overall, and by site and pathogen

	aHR[a] (95% CI)	E-value for the central value of the estimate	E-value for the lower bound of the confidence interval
Serious infections, overall	1.34 (1.32-1.36)	2.01	1.97
**By infection site**			
Digestive tract	1.52 (1.48-1.55)	2.41	2.32
ENT sphere	1.47 (1.41-1.52)	2.30	2.17
Lower respiratory tract	1.22 (1.19-1.25)	1.74	1.67
Kidneys or urinary tract	1.20 (1.15-1.25)	1.69	1.57
Musculosqueletal system	1.17 (1.01-1.37)	1.62	1.11
Nervous system	1.31 (1.11-1.54)	1.95	1.46
**By infection pathogen**			
Bacterial	1.56 (1.50-1.63)	2.49	2.37
Viral	1.30 (1.28-1.33)	1.92	1.88

[a]Cox models adjusted for time-fixed covariates, namely sociodemographic characteristics: age at index date, sex, CMUC, deprivation index, size of the urban unit, calendar year at index date; pregnancy and delivery characteristics: maternal age, ART, maternity status, mode of delivery, gestational age, birth weight; maternal comorbidities: diabetes, hypertension, obesity, tobacco, alcohol, and illicit substance consumption; healthcare use in mothers: preventive medications during pregnancy; healthcare use in children: outpatient physician visits, outpatient pediatrician visits, drug dispensings, hospital stays; and for time-varying covariates, namely season, child comorbidities and drug exposures: respiratory diseases, neurological disease, diabetes, obesity, liver diseases, chronic kidney diseases, cardiovascular diseases, immunosuppression, digestive diseases, chronic corticosteroid treatment, NSAID treatment. For each categorical covariate, missing values, if any, were considered as a separate category.

© 2023 Lassalle M et al. *JAMA Pediatrics.*
