# Supplementary material for: Proton Pump Inhibitor Use and Risk of Serious Infections in Young Children
Source: JAMA Pediatr. 2023 Aug 14;177(10):1028–38. doi: 10.1001/jamapediatrics.2023.2900 (PMC10425862; doi:10.1001/jamapediatrics.2023.2900)
Supplement: Supplement 2. — Data sharing statement [file jamapediatr-e232900-s002.pdf]

Data Sharing Statement

Lassalle. Proton Pump Inhibitor Use and Risk of Serious Infections in Young Children. *JAMA Pediatr*. Published August 14, 2023. doi:10.1001/jamapediatrics.2023.2900

Data
Data available: No
